# Exploring the Common Mechanism of Fungal sRNA Transboundary Regulation of Plants Based on Ensemble Learning Methods

**DOI:** 10.3389/fgene.2022.816478

**Published:** 2022-02-11

**Authors:** Junxia Chi, Hao Zhang, Tianyue Zhang, Enshuang Zhao, Tianheng Zhao, Hengyi Zhao, Shuai Yuan

**Affiliations:** ^1^ College of Software, Jilin University, Changchun, China; ^2^ Key Laboratory of Symbolic Computation and Knowledge Engineering of Ministry of Education, Jilin University, Changchun, China; ^3^ College of Computer Science and Technology, Jilin University, Changchun, China

**Keywords:** Magnaporthe oryzae, Botrytis cinerea, Phytophthora infestans, sRNA, ensemble Learning, cross-plant regulatory commonality

## Abstract

Studies have found that pathogenic fungi and plants have sRNA transboundary regulation mechanisms. However, no researchers have used computer methods to carry out comprehensive studies on whether there is a more remarkable similarity in the transboundary regulation of plants by pathogenic fungi. In this direction, high-throughput non-coding sRNA data of three types of fungi and fungi-infected plants for 72 h were obtained. These include the *Magnaporthe*, *Magnaporthe oryzae* infecting *Oryza sativa*, *Botrytis cinerea*, *Botrytis cinerea* infecting *Solanum lycopersicum*, *Phytophthora infestans* and *Phytophthora infestans* infecting *Solanum tuberosum*. Research on these data to explore the commonness of fungal sRNA transboundary regulation of plants. First, using the big data statistical analysis method, the sRNA whose expression level increased significantly after infection was found as the key sRNA for pathogenicity, including 355 species of *Magnaporthe oryzae*, 399 species of *Botrytis cinerea*, and 426 species of *Phytophthora infestans*. Secondly, the target prediction was performed on the key sRNAs of the above three fungi, and 96, 197, and 112 core nodes were screened out, respectively. After functional enrichment analysis, multiple GO and KEGG_Pathway were obtained. It is found that there are multiple identical GO and KEGG_Pathway that can participate in plant gene expression regulation, metabolism, and other life processes, thereby affecting plant growth, development, reproduction, and response to the external environment. Finally, the characteristics of key pathogenic sRNAs and some non-pathogenic sRNAs are mined and extracted. Five Ensemble learning algorithms of Gradient Boosting Decision Tree, Random Forest, Adaboost, XGBoost, and Light Gradient Boosting Machine are used to construct a binary classification prediction model on the data set. The five indicators of accuracy, recall, precision, F1 score, and AUC were used to compare and analyze the models with the best parameters obtained by training, and it was found that each model performed well. Among them, XGBoost performed very well in the five models, and the AUC of the validation set was 0.86, 0.93, and 0.90. Therefore, this model has a reference value for predicting other fungi’s key sRNAs that transboundary regulation of plants.

## Introduction

Fungal diseases, accounting for 70–80% of plant diseases, can infect each other, have an infestation process, and continue to persecute the growth and development of plants, resulting in a reduction in output and causing substantial economic losses to the world. Among them, fungi such as *Magnaporthe oryzae* and *Phytophthora infestans* are more harmful to food, and fungi such as *Botrytis cinerea* are more harmful to fruits and vegetables. In the development of agriculture for about 10,000 years, humans have been fighting against plant fungal diseases, a hot issue studied for a long time (Dean et al., 2012). Therefore, a more comprehensive understanding of fungi infecting plants and effective prevention and control is significant in reducing various losses.

*Magnaporthe oryzae* is distributed worldwide and occurs at every stage of *Oryza sativa* growth. About 30% of the *Oryza sativa* loss in the world is caused by *Magnaporthe oryzae* every year (Deng et al., 2017), which can feed at least 60 million people (Nalley et al., 2016). At the same time, the fungus can also infect food crops such as Triticum aestivum L, Secale cereale L, millet, Setaria italica and Avena sativa L (Chakraborty et al., 2021; Roy et al., 2021). *L*L*Botrytis cinerea* infects a wide range and can harm more than 470 plants such as Solanaceae, Cucurbitaceae and Rosaceae (Fillinger and Elad, 2016). The pathogen has always been a model for studying the molecular mechanism of the interaction between the host and the pathogen (Liu et al., 2018). Late blight caused by *Phytophthora infestans* is one of the most destructive *Solanum tuberosum* diseases globally and the most important yield-limiting factor in *Solanum tuberosum* production (Haverkort et al., 2016). Effectively preventing and controlling *Magnaporthe oryzae*, *Botrytis cinerea*, *Phytophthora infestans* and other kinds of fungi has always been a hot research issue. Therefore, this article explores the infection mechanism of *Magnaporthe oryzae* to *Oryza sativa*, *Botrytis cinerea* to *Solanum lycopersicum*, and *Phytophthora infestans* to *Solanum tuberosum* from a new perspective. It is particularly important to formulate persistent and broad-spectrum control strategies for *Oryza sativa* resistance to *Magnaporthe oryzae*, *Solanum copersicum* resistance to *Botrytis cinerea*, *Solanum tuberosum* resistance to *Phytophthora infestans*, and even the entire plant kingdom to resist fungi.

sRNA was first discovered in 1993, and more and more researchers are currently studying sRNA and have made a lot of progress Li and Li, 2018). Studies have found that some small double-stranded RNA (dsRNA) can degrade mRNA and cause gene silencing, which is called RNA interference (RNAi) (Guo and Kemphues, 1995; Fire et al., 1998). It is present in all eukaryotic cells, such as fungi, plants, and animal cells. The process is that dsRNA is decomposed into two sRNAs under the action of RNaseIII enzyme, one of which is added to RISC (RNA-induced silencing complex) to inhibit protein production. Studies have found that sRNA can transmit and silence each other’s genes between objects with vitality. This phenomenon is cross-species RNAi (Cai et al., 2018a). Recent studies have shown that fungal sRNA can cross borders into plants and play a regulatory role (Deng et al., 2018; Kusch et al., 2018; Zanini et al., 2021). In 2013, a study confirmed *Botrytis cinerea* transports toxic sRNA utility factors into *Arabidopsis* cells, silences immune-related genes, and successfully verified that the three sRNAs of Bc-siR3.1, Bc-siR3.2 and Bc-siR5 are in *Botrytis cinerea*. Play an active role in the pathogenicity of the disease (Weiberg et al., 2013). When the *Arabidopsis* ago1-27 mutant was infected by *Verticillium dahliae*, it resisted the fungal infection; In *Arabidopsis* ago7-2, dcl4-2, rdr2-4, and rdr6-15 mutants of RNA silencing pathways that are infected, severe symptoms appear (Ursula et al., 2009; Weiberg et al., 2013). A study in 2016 indicated that sRNA effectors produced by *Botrytis cinerea* Dicer protein 1 (BC-DCL1 and BC-DCL2) were delivered to *Solanum lycopersicum* and *Arabidopsis* cells, and the host immune gene was silenced. It also showed that sRNAs targeting BC-DCL1 and BC-DCL2 in *Arabidopsis* and *Solanum lycopersicum* can silence the BC-DCL gene and reduce the pathogenicity and growth of *Botrytis cinerea*. This discovery indicates a bidirectional cross-border RNAi between plants and fungi, and then experimentally verified that the application of genes targeting BC-DCL1 and BC-DCL2 on the surfaces of fruits, vegetables and flowers can significantly inhibit *Botrytis cinerea* disease (Wang et al., 2016). Research by Ming Wang and other researchers in 2018 showed that a small part of the sRNA of *powdery mildew* fungi has targets in plants, indicating that there may be cross-kingdom RNA transfer between *powdery mildew* fungi and their respective plant hosts (Kusch et al., 2018). Cai Q found that *Arabidopsis thaliana* can transport its sRNA into *Botrytis cinerea* tissues. By inhibiting related genes of *Botrytis cinerea*, it prevents and resists the pathogenicity of *Botrytis cinerea* (Cai et al., 2018b). In 2019, researchers sequenced the sRNA of *Sclerotinia sclerotiorum in vitro* and infecting *Arabidopsis thaliana* respectively, and found that at least 374 distinct highly abundant sRNAs were produced during the infection process. Target prediction was performed and it was found that the targets were enriched in functional domains related to plant immunity (Derbyshire et al., 2019). The above findings lay the foundation and broaden ideas for an in-depth discussion of plant fungi. This article assumes that the transboundary regulation of plants and fungi is widespread.

With the wide application of extensive biological data analysis that has achieved good results in the biological field, and the sRNA data of fungi and plants are becoming more and more perfect, this provides a basis for the cross-border research of fungi and plants sRNA. Some researchers used the SVM model to predict the key sRNA of *Magnaporthe oryzae* (Zhang et al., 2019). Some researchers used the Random Forest model to predict the key sRNA of *Phytophthora infestan* (Liu et al., 2020). Some researchers used multiple machine learning models to predict the key sRNAs of *Phytophthora infestans and Magnaporthe oryzae* pathogenicity (Zhang et al., 2020). The researchers have proved that machine learning models have excellent effects on predicting the pathogenicity of key sRNAs in fungi, especially the Random Forest and AdaBoost models, both of which belong to Ensemble learning.

However, they only classify and predict one or two kinds of fungi, and the prediction models used for each type of fungi are inconsistent. For this reason, this paper uses a variety of ensemble learning models to predict the pathogenic key sRNAs of three kinds of fungi with huge differences to find a generic model. In this paper, we studies the sRNA data before and after *Magnaporthe oryzae* infects the *Oryza sativa*, *Botrytis cinerea* infects the *Solanum lycopersicum*, and *Phytophthora infestans* infects the *Solanum tuberosum*. The key sRNAs in the infection process were mined through data analysis, and functional enrichment analysis was performed on them to find the functional commonality of the three fungal sRNAs in the transboundary regulation of plants. At the same time, five ensemble learning methods are used to construct a binary classification prediction model, and five indicators are used to evaluate the model to select the optimal model. Thus, this article provides a reference for selecting key sRNA algorithm models for predicting fungal pathogenicity, provides a direction for the study of other fungal sRNAs transboundary regulation of plants, and provides a theoretical basis and new ideas for the prevention and control of plant fungal diseases.

## Data and Methods

**FIGURE 1:**
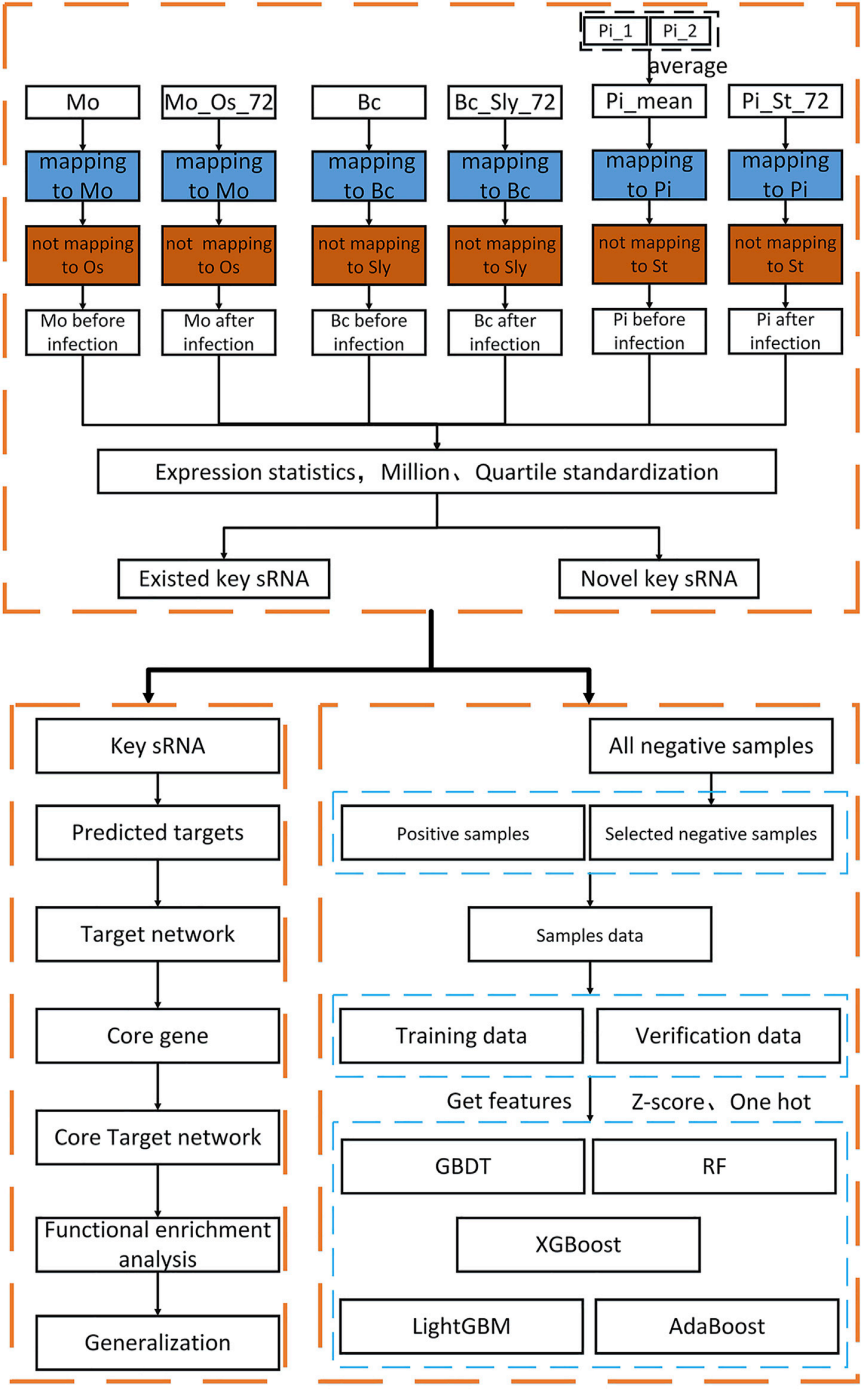
Overall research flow chart. In the first and fourth rows, *Mo* is the abbreviation of *Magnaporthe oryzae* sRNA, *MO_Os_72* is the abbreviation after *Magnaporthe oryzae* sRNA infecting *Oryza sativa* 72 h, *Bc* is the abbreviation of *Botrytis cinerea* sRNA, *Bc_Sly_72* is the abbreviation after *Botrytis cinerea sRNA* infecting *Solanum lycopersicum* 72 h, *Pi* is the abbreviation of *Phytophthora infestans* sRNA, *Pi_St_72* is the abbreviation after *Phytophthora infestans sRNA* infecting *Solanum tuberosum* 72 h. In the second and third rows are the corresponding genomes.

This article uses multiple databases to obtain all the relevant data of three fungi (*Magnaporthe oryzae*, *Botrytis cinerea*, *Phytophthora infestans*) and three plants (*Oryza sativa*, *Solanum lycopersicum*, *Phytophthora infestans*). Taking the changes of fungi before and after infecting plants as the research direction, the data obtained are analyzed and the key sRNA sequences that cause disease are screened. The overall process of this article is shown in Figure 1.

### Data Source and Preprocessing

#### Data Sources

Seven kinds of data were obtained from GEO database (https://www.ncbi.nlm.nih.gov/geo/), including: *Magnaporthe oryzae* mycelium SRNA data set (GSM1059882), *Magnaporthe oryzae* mycelium infected *Oryza sativa* leaves 72 h mixed data set (GSM1059888); *Botrytis cinerea* mycelium sRNA data set (GSM1101910), 72-h mixed data set of *Botrytis cinerea* mycelium infecting *Solanum lycopersicum* leaves (GSM1101915); Data set of *Phytophthora infestans* rep1 sRNA (GSM1212963) and rep2 sRNA (GSM1212964), *Phytophthora infestans* infected *Solanum tuberosum* leaf tissue 72 h mixed data set (GSM1545158). Eight kinds of data were obtained from NCBI, including: *Magnaporthe oryzae* genome data (Pyricularia oryzae 70-15 (assembly MG8)) and *Oryza sativa* genome data (Oryza sativa Japonica Group (assembly IRGSP-1.0)), *Botrytis cinerea* genome data (Botrytis cinerea B05.10 (assembly ASM14353v4)) and *Solanum lycopersicum* genome data (Solanum lycopersicum (assembly SL3.0)) and *Phytophthora infestans* genome data (AATU01) and *Solanum tuberosum* genome data (AEWC01) and *Oryza sativa* mRNA data (https://www.ncbi.nlm.nih.gov/nuccore) and *Solanum lycopersicum* gene annotation (https://www.ncbi.nlm.nih.gov/assembly/GCF_000188115.4). Obtain the *Solanum lycopersicum* mRNA data (SL2.5) from the Ensemble plant database (http://plants.ensembl.org/index.html). Obtain the *Solanum tuberosum* transcript data (PGSC_DM_v3.4_Transcript-UPDATE) and genome annotation data (PGSC_DM_v3.4_gene) from the SPUD database (http://solanaceae.plantbiology.msu.edu/index.shtml).

#### Adapter, Quality Information, Length Processing

**FIGURE 2:**
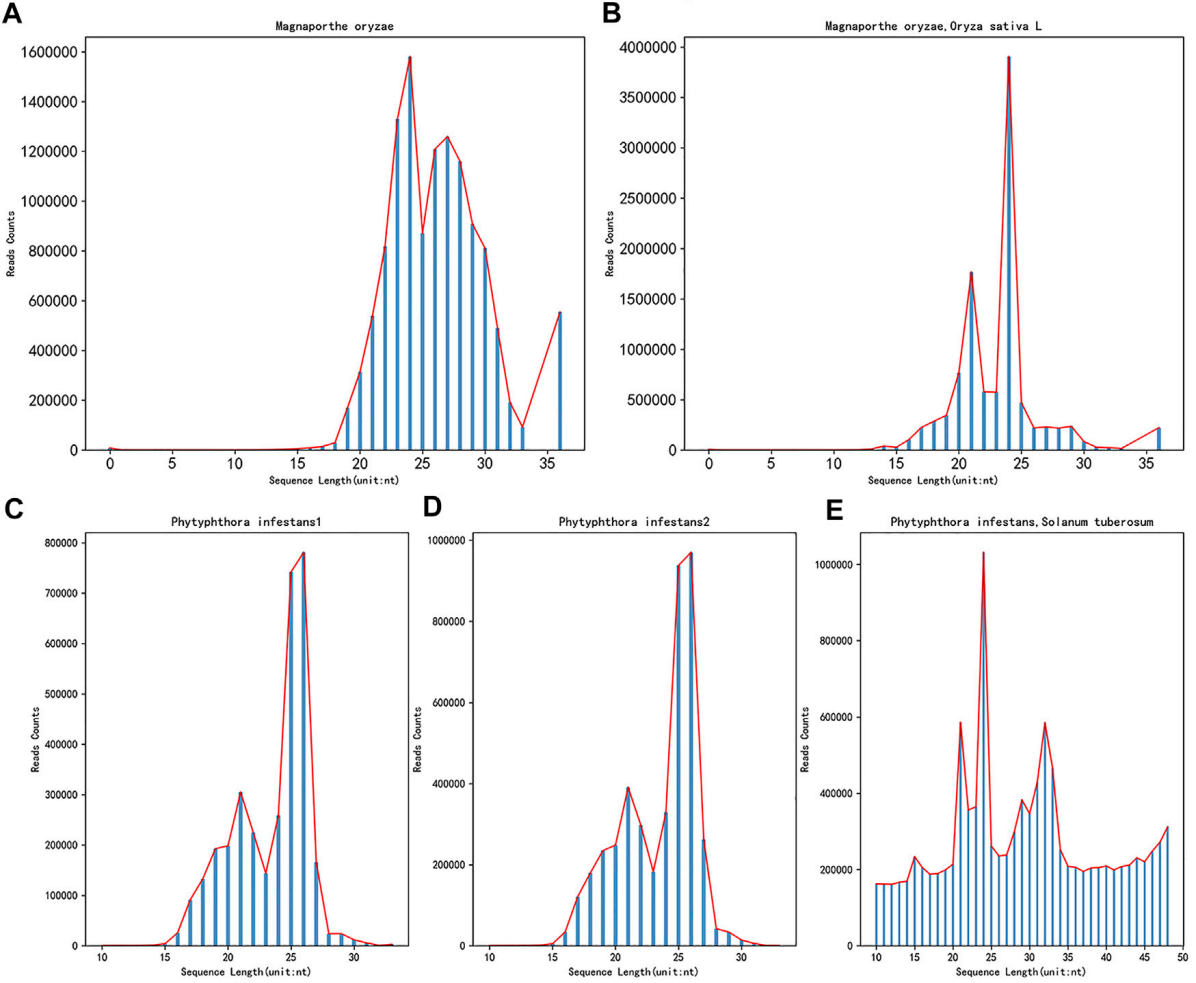
The length distribution diagram after removing the adapters before and after the infection. **(A)** shows the sRNA data of *Magnaporthe oryzae* after removing the adapters, **(B)** shows the sRNA data of *Magnaporthe oryzae* infected with *Oryza sativa* for 72 h after removing the adapters. **(C)** shows the sRNA data of *Phytophthora infestans* rep1 after removing the adapter, **(D)** shows the sRNA data of *Phytophthora infestans* rep2 after removing the adapter, **(E)** shows the sRNA data of *Phytophthora infestans* infected with *Solanum tuberosum* for 72 h after removing the adapters. The abscissa in the figure represents the length of the sequence, and the ordinate represents the number corresponding to the length of the sequence.

After obtaining the data needed for the research, it was found that the data format obtained from the GEO database was in SRA format. To facilitate subsequent operations, the Sratoolkit tool (https://hpc.nih.gov/apps/sratoolkit.html) was used to convert it into the standard fastq format. Since the obtained sRNA data is high-throughput sequencing, removing the adapters from the sequence is necessary. This article uses Cutadapt (https://cutadapt.readthedocs.io/en/stable/) to remove the adapters. Due to the obtained data of *Botrytis cinerea* and the 72-h data of *Botrytis cinerea* infecting *Solanum lycopersicum*, the process of removing adapters and masking low-quality and low-complexity sequences has been carried out. Therefore, this article will no longer perform the processing mentioned earlier on these two kinds of data, only controlling the length. After the data is removed adapters, the sRNA data of the two fungi and the 72 h mixed data of the corresponding plant of the fungus infection, the length and the number of sequences corresponding to the length are distributed as shown in Figure 2.

The source article (Raman et al., 2013) of the *Magnaporthe oryzae* data and the mixed data of the *Magnaporthe oryzae* infecting *Oryza sativa* for 72 h, there is no quality information processing operation (Raman et al., 2013). Therefore, this article does not perform quality control operations on it, only performs quality control operations on the data of *Phytophthora infestans* and the mixed data of the 72 h of *Phytophthora infestans* infecting *Solanum tuberosum*. Use fastQC tools (http://www.bioinformatics.babraham.ac.uk/projects/fastqc/) for quality control, and set at least 80% of the bases in each sRNA sequence to have a quality value greater than or equal to 33. In addition, this article only studies base sequences with a length of 18–25, so it is necessary to control the length and only keep the sRNA sequences that match the length.

After the above processing, it is necessary to perform sequence expression statistics on the six kinds of sRNA data respectively. The specific method is: deduplicate the data to obtain the sequence type, use the data sequence after deduplication, and count the number of occurrences of each sequence in the data file before deduplication is the expression level of the sequence. The statistical results of the number of six sRNA types (not the number) are shown in Table 1.

**TABLE 1:** Data volume statistics before and after removing adapters, length and quality control.

Raw data	After adapter (not deduplicated)	Length and quality control (deduplicated)
*Magnaporthe oryzae*	12376438	350381
*Oryza sativa*, 72 hpi (*Magnaporthe oryzae*)	103911039	1208229
*Botrytis cinerea*		584047
*Solanum lycopersicum*, 72 hpi (*Botrytis cinerea*)		812186
*Phytophthora infestans* _1	3341758	1027945
*Phytophthora infestans* _2	4296476	1214340
*Phytophthora infestans* _mean		1879202
*Solanum tuberosum*, 72 hpi (*Phytophthora infestans*)	11016347	1077596

#### sRNA Sequence Mapping to the Genome

**FIGURE3:**
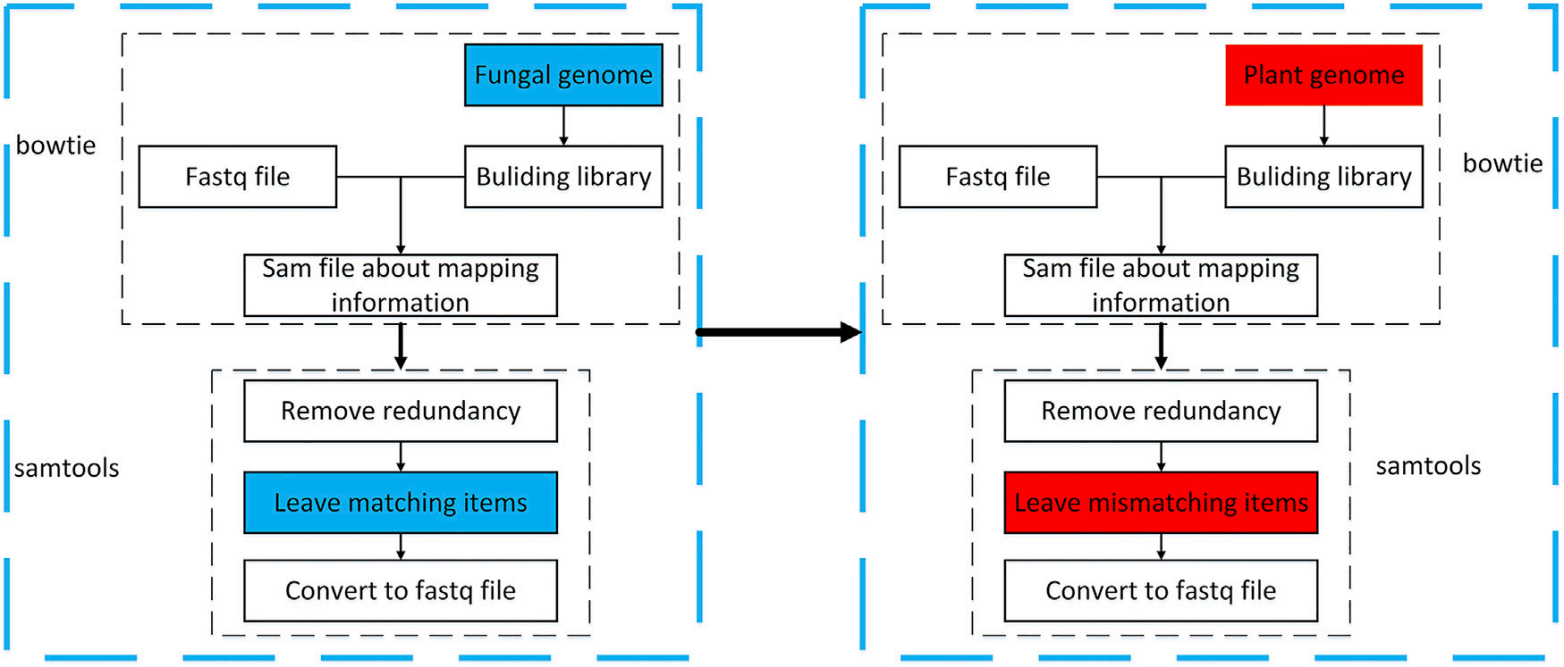
Flow chart of sRNA sequence mapping to genome.

As this article studies fungal sRNA transboundary regulation of plants, it is necessary to find sRNA sequences with apparent changes in expression before and after fungal infection of plants. However, and the data obtained in the previous step, we cannot guarantee that it is a completely fungal sRNA sequence, it may contain pollution and plant sRNA sequence. Therefore, this study uses the tools bowtie2 (https://sourceforge.net/projects/bowtie-bio/files/bowtie2/) (the short sequence is mapped to the genome, set strict matching, and the mismatch parameter is 0) and samtools (http://www.htslib.org/download/) (operation sam and bam toolset) to map sequences to fungal and plant genomes. Sequences that match the fungal genome and those that do not match the plant genome are retained. The operation process is shown in Figure 3.

Among them, *Phytophthora infestans* rep1, rep2, after removing adapters, quality information and length control but not yet mapped to the genome, the number of sequence types are 1,027,945 and 1,214,340, respectively. For the convenience of the next work, this article mixes the two data sets and then performs the mapping operation. The average expression is used as the subsequent data of this experiment. The number of sequence types obtained is 1,879,202, which is quite different. So, it makes sense for us to select two repeated experiments for mixing. After the mapping, the number of sequence types of the sixsRNA data is counted, and the statistical results are shown in Table 2.

**TABLE 2:** Data volume statistics after Map.

Library	sRNA species mapped to the fungus genome	Species after removal of plants sRNA
*Magnaporthe oryzae*	87443	87299
*Oryza sativa*, 72 hpi (*Magnaporthe oryzae*)	11194	10981
*Botrytis cinerea*	306783	16553
*Solanum lycopersicum*, 72 hpi (*Botrytis cinerea*)	21550	812186
*Phytophthora infestans*	1128507	1121519
*Solanum tuberosum*, 72 hpi (*Phytophthora infestans*)	91925	87068

### Standardization of sRNA Expression and Selection of Key sRNA

#### Standardization of Expression

It is found from Table 2 that the number of sRNA types and expression levels of the three fungi before and after infection have undergone tremendous changes. In order to avoid errors in obtaining the sRNA sequence whose expression level is significantly higher after infection than before infection, it is necessary to standardize the expression level to make it comparable. This paper uses two methods to standardize the data. First, the million-standardization method is used for the data after removing the adapters, quality information and length control. Second, the specific calculation method is denoted in Eq. 1:

$$x^{ii}=\frac{100w\times x^{i}}{sum^{i}}$$ (1)

Where $x^{ii}$ is the count of each sRNA sequence after normalization, $x^{i}$ is the expression level of each sRNA sequence before normalization, and $sum^{i}$ is the sum of the expression level of all sRNA sequences. On this basis, the quartile standardization method is used for the data mapped to the genome (fungi and plant). First, sort the expression levels of sRNA sequences in descending order, select the expression levels at each 3/4 position as a reference value, and convert them into corresponding multiples.

#### Select Key sRNA

**FIGURE 4:**
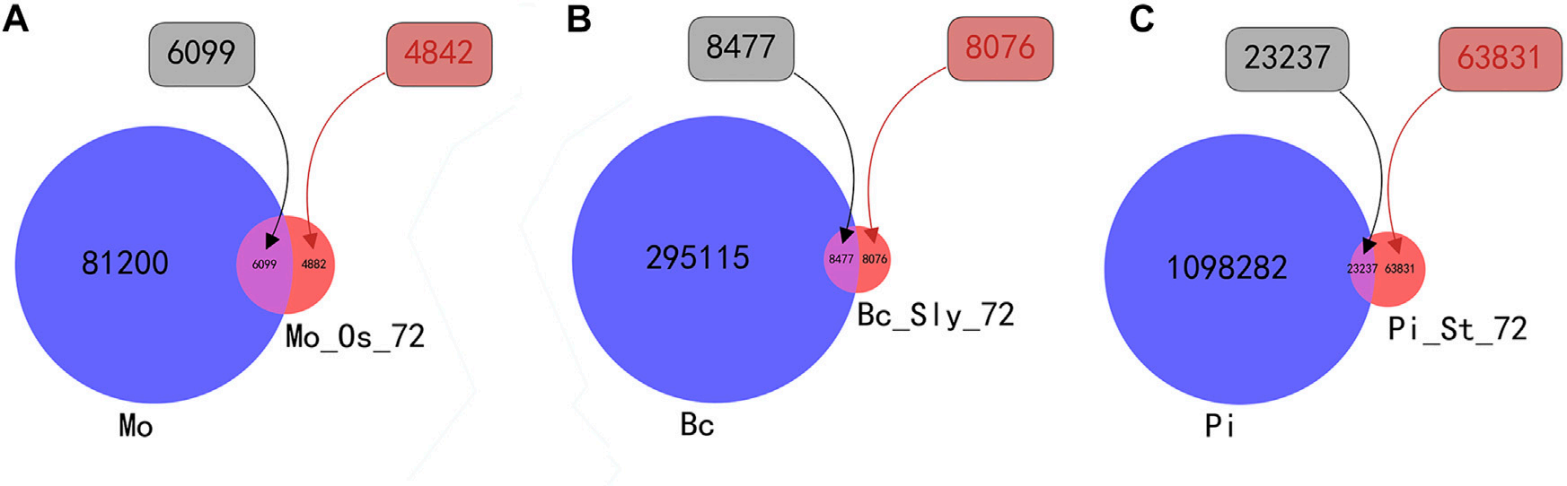
Venn diagram of the number of sRNA types before and after infection. *Mo* is the abbreviation for the number of *Magnaporthe oryzae* sRNA species, and *MO_Os_72* is the abbreviation for the number of *Magnaporthe oryzae* sRNA species after *Magnaporthe oryzae* infecting *Oryza sativa* for 72 h. *Bc* is the abbreviation for the number of *Botrytis cinerea* sRNA species, and *Bc_Sly_72* is the abbreviation for the number of *Botrytis cinerea* sRNA species after *Botrytis cinerea* infecting *Solanum lycopersicum* for 72 h. *Pi* is the abbreviation for the number of *Phytophthora infestans* sRNA species, and *MO_Os_72* is the abbreviation for the number of *Phytophthora infestans* sRNA species after *Phytophthora infestans* infecting *Solanum tuberosum* for 72 h. **(A)** shows the intersection and complementary set of the number of sRNA species before and after *Magnaporthe oryzae infects Oryza sativa*. **(B)** shows the intersection and complementary set of the number of sRNA species before and after *Botrytis cinerea* infects *Solanum lycopersicum*. **(C)** shows the intersection and complementary set of the number of sRNA species before and after *Phytophthora infestans* infects *Solanum tuberosum*.

Based on the statistics of sRNA sequences, there are 87299 species of *Magnaporthe oryzae* before infection, and 10,981 species after 72 h of infection. Among them, there are 6,099 species of sequences that coexist before and after infection. There are 4,882 newly generated sequences after infection; there are 303,592 species of *Botrytis cinerea* and 16553 species after 72 h of infection. Among them, 8,477 species of data coexist before and after infection, and there are 8,076 newly generated sequences after infection; there are 1,121,519 species of *Phytophthora infestans* before infection, and sequences 87068 species after 72 h of infection. There are 23237 species of sequences that coexist before and after infection, and there are 63831 newly generated sequences after infection; For the convenience of viewing, the statistical results are shown in Figure 4.

The above figure shows that after the fungus infects the plant, the number of types of sRNA sequences is significantly reduced. This article believes that after standardizing the sRNA sequence, the expression level of fungi after infection is significantly increased compared with that before infection, that is, the sRNA sequence with a fold increase in the expression level after infection relative to the pre-infection and higher expression level after infection is the key sRNA sequence for fungal transboundary regulation of plants. This article is divided into two parts to study the sRNA sequences whose expression levels increase significantly after infection. The first part is the sRNA sequences that co-exist before and after infection with significantly increased expression levels. The specific calculation method is denoted in Eq. 2:

$$R=\frac{count\;of\;reads\;after\;infection-count\;of\;reads\;before\;infection}{count\;of\;reads\;before\;infection}$$ (2)

Among them, *R* is the growth rate after infection relative to before infection, count of reads after infection is the expression level of a certain sRNA sequence after infection, and count of reads before infection is the expression level of the corresponding sequence before infection.

**FIGURE 5:**
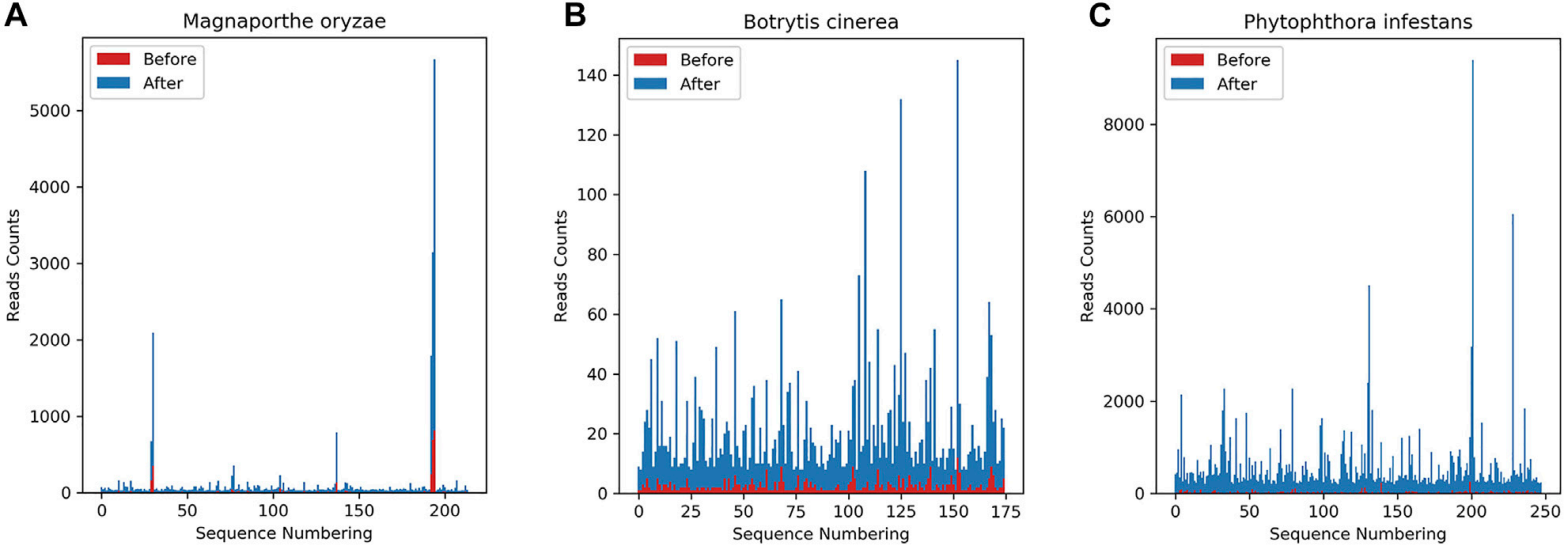
Differences in sRNA expression levels co-existing before and after infection. The abscissa is the sequence index, sorted according to the sequence dictionary order, and the ordinate is the amount of expression. Red indicates the expression level before fungus infection, and blue indicates the corresponding expression level after fungus infection. It can be seen from the figure that there is a significant difference in expression before and after infection. **(A)** shows the number of sRNA species co-existing before and after *Magnaporthe oryzae* infects *Oryza sativa* and the expression level of each sRNA. **(B)** shows the number of sRNA species co-existing before and after *Botrytis cinerea* infects *Solanum lycopersicum* and the expression level of each sRNA. **(C)** shows the number of sRNA species co-existing before and after *Phytophthora infestans* infects *Solanum tuberosum* and the expression level of each sRNA.

The key sRNAs in this paper are based on both the growth rate and the expression level, and the relevant data after the infection is screened. Among them, *Magnaporthe oryzae* was a sequence co-existing before and after infection and met the two conditions of growth rate greater than 2 and expression level greater than or equal to 25, and 214 sequences were obtained; *Botrytis cinerea* is a sequence co-existing before and after infection and meeting the two conditions of growth rate greater than or equal to 2 and expression level greater than or equal to 7, resulting in 175 sequences; *Phytophthora infestans* is a sequence coexisting before and after infection, and simultaneously meets the two conditions of growth rate greater than 2 and expression level greater than or equal to 200, resulting in 248 sequences. The differences in the expression levels of the three fungal sRNAs before and after infection are shown in Figure 5.

The second part of the key sRNA is the newly produced sRNA sequence after the fungus infects the plant. For *Magnaporthe oryzae*, 141 with expression level greater than or equal to 30 is selected, for *Phytophthora infestans*, 178 with expression level greater than or equal to 60, and for *Botrytis cinerea* 224 with expression greater than or equal to 18. The statistical results are shown in Table 3.

**TABLE 3:** Key sRNA data volume statistics.

Data	Key sRNA	Key_inner	Key_outer
*Oryza sativa*, 72 hpi (*Magnaporthe oryzae*)	355	214	141
*Solanum lycopersicum*, 72 hpi (*Botrytis cinerea*)	399	175	224
*Solanum tuberosum*, 72 hpi (*Phytophthora infestans*)	426	248	178

In this paper, 355 species of *Magnaporthe oryzae*, 399 species of *Botrytis cinerea*, and 426 species of *Phytophthora infestans* selected from the above two parts were used as key sRNAs for subsequent processing.

### Target Gene Prediction and Select the Core Node

Based on the three types of key sRNA sequences screened out above, in order to find the relevant biological processes and regulatory pathways in the cross-border regulation of the three fungal sRNAs, target predictions were performed on the three key sRNAs. This article uses the Tapir tool (http://bioinformatics.psb.ugent.be/webtools/tapir/) to make predictions, converts the files storing key sRNA sequences into fasta format using codes, replaces all T with U. Then use the mRNA data of *Oryza sativa*, the CDS data of *Solanum lycopersicum*, and the transcript data of *Solanum tuberosum* respectively for target prediction. After the target is predicted, the identifier is extracted from the result file by programming. The identifier can be matched to the corresponding gene ID or protein ID from the mRNA, transcript, CDS, or gene annotation file. In this paper, the *Oryza sativa* mRNA data file contains its gene ID; the *Solanum lycopersicum* CDS file also contains its gene ID; the *Solanum tuberosum* transcript file has neither gene ID nor protein ID. This article uses gene annotation files to match the corresponding gene ID. The above matching process needs to be programmed to achieve. After matching, functional analysis can be performed in a variety of tools. This study uses the String online tool (https://www.string-db.org/cgi/input?sessionId=btV3GVJkiL7j&input_page_show_search=on).

The corresponding relationship between sRNA and mRNA or CDS or transcript is 1 to 0 to multiple. After running the target prediction on the server, the number of key sRNA types of the three fungi, the number of targeted mRNA/CDS/Transcript, the number of types after deduplication, the number of genes targeted for plants, the type of gene ID after deduplication, and related network PPI enrichment *p*-value are counted, as shown in Table 4.

**TABLE 4:** Key sRNA targeting statistics.

Target	sRNA	mRNA/CDS/Transcript	Deduplication	Gene	Gene deduplication	PPI enrichment *p*-value
*Magnaporthe oryzae*	355	2,860	2,539	2,539	1,657	0.765
*Botrytis cinerea*	399	2,704	1975	1975	1975	0.291
*Phytophthora infestans*	426	3,325	2,221	2,221	1,519	7.15e-14

From the above Table, it can be found that the target prediction results of the three fungi targeting plants are relatively large, and the *p*-value is significant, resulting in a vast network, and obvious pathway enrichment is not easy to be found. Therefore, we need to screen the core nodes separately to find obvious enrichment functions and regulatory pathways.

In this paper, the core nodes are screened in two steps. The first step is to change the confidence between nodes in the network. The default confidence in String is 0.400, and we customize it to 0.600, which has higher confidence. After updating the set confidence, export the tsv file in the String database. The tsv file stores the two protein nodes that interact with each other, the annotations of the two nodes, and the corresponding confidence.

The second step is based on the first step to filter by the size of the node degree. This paper controls the degree of the node through programming, repeatedly importing it into the String database to observe the effect, and finally selects the node of the appropriate degree. Among them, *Oryza sativa* retains nodes with a degree greater than or equal to 5 and obtains 96 core nodes, as shown in Supplementary Table S1; *Solanum lycopersicum* retains nodes with a degree greater than or equal to 8 and obtains 197 core nodes, as shown in Supplementary Table S2; *Solanum tuberosum* retains nodes with a degree greater than or equal to 5 and obtains 112 core nodes, as shown in Supplementary Table S3.

### Predictive Model Construction

#### Negative Sample Selection

This article uses the three key sRNAs mined in 2.2.2 as three sets of positive sample data sets. After removing the positive samples from the infected sequence, it is found that the number of positive samples and non-positive samples of the three fungi is very different. There will be a serious imbalance problem if these samples are directly regarded as negative samples, so negative samples need to be selected. This article first analyzes the length distribution of the sequence in all negative samples and the statistics of the expression of each length. It then extracts each length separately to ensure that the extracted negative sample sequence has the same proportions as all non-positive samples. Finally, the uniform sorting method in the roulette method is used to extract negative samples with a ratio of positive and negative samples of 1:4. To facilitate viewing, the number of sRNAs, the number of non-positive samples, the ratio of positive samples to non-positive samples, the number of negative samples, and the number of positive and negative samples of the three fungi are counted, as shown in Table 5.

**TABLE 5:** Statistics of positive and negative samples.

Sample	Positive sample	Non-positive sample	Proportion	Negative samples	Positive and negative samples
*Magnaporthe oryzae*	355	10626	1:30	1,420	1,775
*Botrytis cinerea*	399	16154	1:40	1,596	1995
*Phytophthora infestans*	426	86642	1:203	1,704	2,130

#### sRNA Feature Extraction and Processing

Before using machine learning to build a model, it is necessary to extract the feature vectors required by the model. Based on the previous sRNA research, this article extracts the features of the sRNA sequences of the three fungi, including 25 base positions, sRNA sequence length, and GC content, Minimum free energy, 5′-end single base, 5′-end double base, 3′-end single base, 3′-end double base and 84 Motif frequency (1-3nt), a total of 116 Features. If the sequence length is less than 25, use N to fill in the vacant bases for base positions. The minimum free energy is obtained in this article using the RNAfold tool (http://rna.tbi.univie.ac.at/cgi-bin/RNAWebSuite/RNAfold.cgi). The input is the fasta format of the positive and negative sample set, and the corresponding sRNA sequence and minimum free energy are output. The output sRNA sequence is not in the format we need, and it needs to be converted through programming. In this paper, when constructing the model, it is found that when the features are added with 5′-end single base and 3′-end single base, the classification effect is better than only 5′-end double base and 3′-end double base, so extract these four features. Motif frequency (1-3nt): 1nt includes 4 types of features, counting the number of occurrences of A, T, G, and C in each sequence; 2nt includes 16 types of features, 2nt includes 16 types of features, counting the number of occurrences of AA, AT, etc in each sequence; 3nt includes 64 types of features, counting the number of occurrences of AAA, AAT, etc in each sequence; Since the constructed model requires digital features, it is necessary to encode the sequence features by onehot. For N, A, C, G, and T, perform four-bit sequential encoding, and for AA (00010001), AC (00010010), … , TT (10001000) perform eight-bit encoding.

After encoding, the base positions are changed from the original 25 features to 100 after encoding. The sequence length, GC%, and MFE remain unchanged. 5′-end single base and 3′-end single base are changed from the original One feature becomes the encoded 4 features, 5′-end double base and 3′-end double base are changed from the original one feature to the encoded eight features. The number of 84 features of the Motif frequency (1-3nt) remains unchanged, and eventually, the original 116 features become the encoded 211 features. There are 3 continuous features, 208 discrete features, and there is a big gap between the continuous feature values. In order to make all data with different magnitudes into the same magnitude and to ensure that the data is comparable, the “Z_Score” method is used to standardize all features. The specific calculation method is denoted in Eq. 3:

$$Feature^{'}=\frac{Feature-\mu }{\sigma }$$ (3)

After the features are standardized, redundant features are removed by identifying high-relevance features, zero-importance features, and low-importance features. Among them, 54 are *Magnaporthe oryzae*, 39 are *Botrytis cinerea*, 32 are *Phytophthora infestans*. The remaining 157 characteristics of *Magnaporthe oryzae*, 172 characteristics of *Botrytis cinerea*, and 179 characteristics of *Phytophthora infestans* were used for subsequent model construction.

#### Model Cross-Validation to Select Optimal Parameters

In this paper, the samples of the three fungi are divided into verification data and training data according to 1:3. To construct a binary classification model, use five ensemble learning algorithms: GBDT (Gradient Boosting Decision Tree), AdaBoost, Random Forest, XGBoost, and LightGBM (Light Gradient Boosting Machine). Then, through the combination of network search and cross-validation, the five classification models of the three data sets are separately trained with parameters. As a result, each data set model has the optimal effect. For the binary classification, there will be deviations between the model prediction and the actual situation, so this article uses four indicators of accuracy, recall, precision, and F1 Score to analyze it. The specific calculation is denoted in Eqs 4–7:

$$Accuracy=\frac{TP+TN}{TP+TN+FP+FN}$$ (4)

$$Precision=\frac{TP}{TP+FP}$$ (5)

$$Recall=\frac{TP}{TP+FN}$$ (6)

$$F1\;Score=\frac{2TP}{2TP+FP+FN}$$ (7)

Among them, T refers to True, F refers to False; P refers to the positive sample, that is, the key sRNA data set of each type of fungus in this article, and N refers to the negative sample, that is, the corresponding part of the non-key sRNA data set obtained by screening using the roulette method. So TP is the correct number of key sRNA for each type of data in the three types of fungi data set, TN is the number of correct non-key sRNA for each type of fungus data set; FN is the number of pathogenic key sRNAs in each type of data set that were incorrectly classified into corresponding fungal non-critical sRNAs, and FP is the number of non-critical sRNAs in each type of data set that were classified into critical sRNAs.

This article takes accuracy as the main evaluation index, recall rate, precision and F1 score as additional evaluation indexes and uses a combination of grid search and cross-validation to train the parameters. The parameters are trained using a combination of grid search and cross-validation. The results obtained after training are shown in Table 6.

**TABLE 6:** The optimal parameters obtained from the training of the five models.

Ensemble Learning	Parameter	*Magnaporthe oryzae*	*Botrytis cinerea*	*Phytophthora infestans*
Gradient Boosting Decision Tree	n_estimators	20	49	95
	max_depth	3	5	3
	learning_rate	0.1	0.1	0.1
	max_features	auto	sqrt	auto
	subsample	0.6	0.8	0.8
Random forest	n_estimators	50	50	68
	max_depth	6	7	9
	criterion	gini	entropy	entropy
	max_features	sqrt	auto	auto
	oob_score	false	false	false
Adaboost	n_estimators	60	60	60
	max_depth	3	4	3
	learning_rate	0.03	0.1	0.08
	criterion	entropy	entropy	entropy
	max_features	auto	auto	auto
XGBoost	n_estimators	20	60	50
	max_depth	3	6	3
	subsample	0.5	0.8	0.6
	gamma	3	4	3
	min_child_weight	5	4	1
Light Gradient Boosting Machine	n_estimators	45	55	58
	max_depth	3	5	5
	learning_rate	0.1	0.1	0.1
	num_leaves	5	9	18
	bagging_fraction	0.6	0.6	0.6

## Results

### Functional Enrichment Analysis

Import the 96, 197, and 112 core nodes selected in 2.3 into the String database again, select the corresponding plants and perform a mapping search. The results show that the overall PPI enrichment *p*-value of the *Oryza sativa* network is 9.66e-15, and the overall PPI enrichment p of the *Solanum lycopersicum* network -value is less than 1.0e-16, and the overall PPI enrichment *p*-value of the *Solanum tuberosum* network is less than 1.0e-16, both of which have high accuracy.

In the network enrichment pathway results, it was found that *Oryza sativa* has 83 Biological Process (Gene Ontology), 25 Molecular Function (Gene Ontology), 16 Cellular Component (Gene Ontology), and 25 KEGG_Pathway. It is found that *Solanum lycopersicum* has 5 Molecular Function and 29 KEGG_Pathways. It is found that there are 26 KEGG_Pathways in *Solanum tuberosum*. The above results False discovery rate are all <0.05. The above GO and KEGG_Pathway can be shown in Supplementary file Supplementary Tables S4–S8.

**FIGURE 6:**
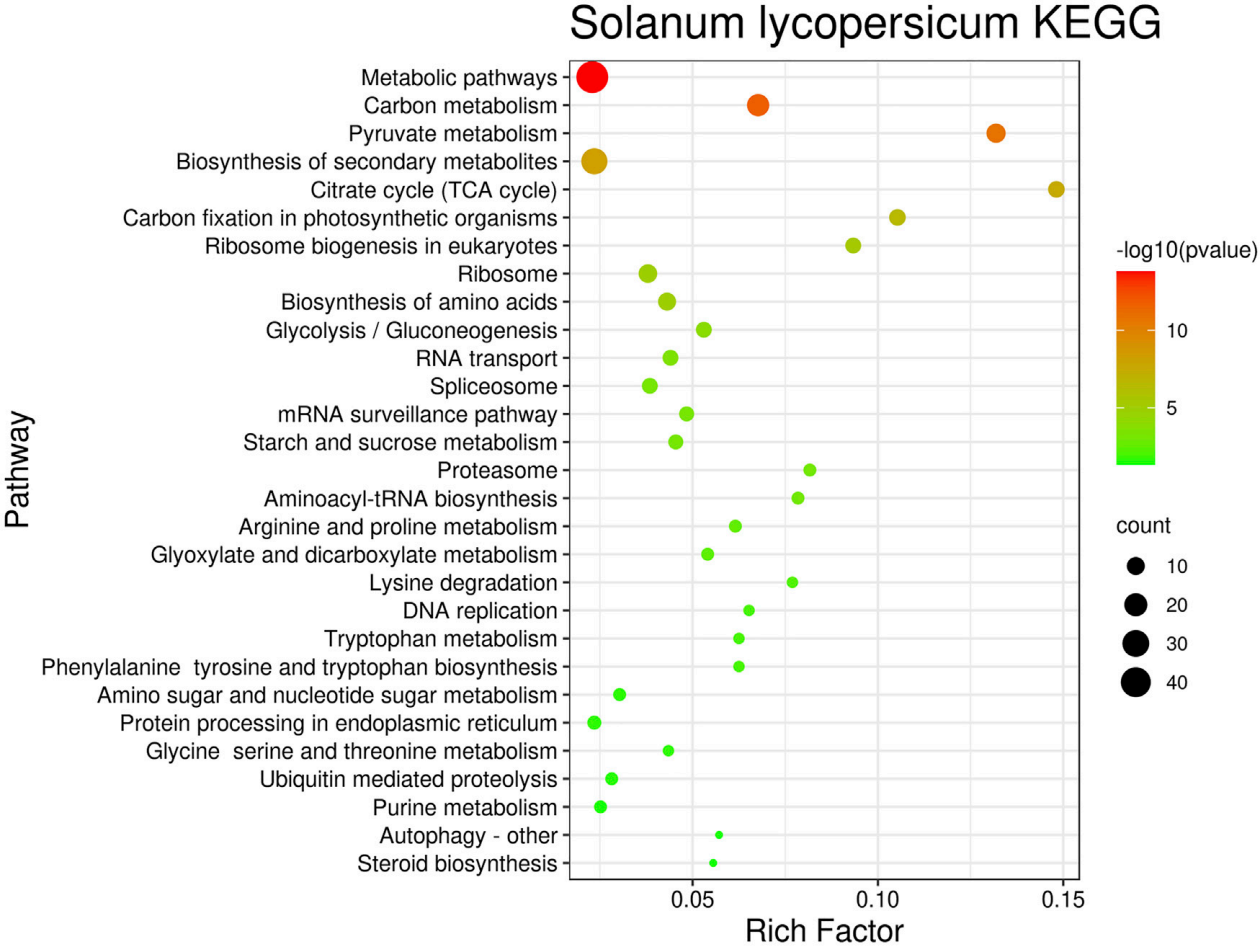
Bubble diagram of *Botrytis cinerea* targeting *Solanum lycopersicum*’s KEGG_Pathway.

In order to analyze these regulatory pathways more intuitively, this article plots the number of genes contained in the regulatory pathways and the reliability of the pathways and enrichment factors in the bubbles figures. The bubble plots corresponding to *Botrytis cinerea* is shown in Figure 6. Bubble plots for *Magnaporthe oryzae* and *Phytophthora infestans* are shown in Supplementary Figures S1, S2 in Supplementary file. Take the negative logarithm-log_10 (*p*_value) processing to the false discovery rate. −log_10 (*p*_value) is proportional to the credibility of the pathway, and the number of genes is proportional to the pathway effect.

### Commonization of Enrichment Pathways

According to the statistical analysis of 3.1, the intersection of Molecular Function (GO) targeting *Oryza sativa* (25 items) and targeting *Solanum lycopersicum* (5 items) is 3; the intersection of KEGG_Pathway targeting *Oryza sativa* (25 items) and targeting *Solanum lycopersicum* (29 items) is 16; The intersection of KEGG_Pathway targeting *Solanum tuberosum* (26) and *Oryza sativa* (25) is 11; the intersection of KEGG_Pathway targeting *Solanum tuberosum* (26) and *Solanum lycopersicum* (29) is 15.

The Molecular Function (GO) targeting *Oryza sativa* and targeting *Solanum lycopersicum* are displayed according to the process ID (process description, false discovery rate of *Magnaporthe oryzae*, false discovery rate of *Botrytis cinerea*), followed by GO:0005524 (ATP binding, 0.0215, 0.0074), GO:0008144 (drug combination, 0.0273, 0.0112), GO:0016772 (transferase activity to transfer phosphorus-containing groups, 0.0328, 0.0135). The false discovery rate is the proportion of false in all discoveries. The smaller the value, the better.

For the KEGG_Pathway targeting *Oryza sativa* and *Solanum lycopersicum*, display it from bottom to top according to the description of the pathway (Magnetic blast fungus false discovery rate, *Botrytis cinerea* false discovery rate), In order Ribosome (1.30E-06,1.11E-05), Carbon metabolism (1.11E-05, 1.87E-12), Ribosome biogenesis in eukaryotes (6.25E-05, 4.26E-06), Glycolysis/Gluconeogenesis (0.00038, 0.0001), Ubiquitin mediated proteolysis (0.0021, 0.0252), Arginine and proline metabolism (0.0026, 0.0026), Citrate cycle (TCA cycle) (0.003, 3.19E-08), Aminoacyl-tRNA biosynthesis (0.003, 0.0011), biosynthesis of secondary metabolites (0.0049, 5.66E-09), Pyruvate metabolism (0.0062, 1.67E-11), Lysine degradation (0.0128, 0.0062), Tryptophan metabolism (0.0156, 0.0098), Phenylalanine tyrosine and tryptophan biosynthesis (0.0191, 0.0098), Spliceosome (0.0291, 0.00057), Metabolic pathways (0.0322, 1.60E-14), Carbon fixation in photosynthetic organisms (0.0355, 3.08E-07).

Regulation of gene expression is essential for plant growth and development. Metabolism is the general term for a series of chemical reactions that maintain plant life, enabling plants to grow and reproduce, maintain structure, and respond to the external environment (Carrington and Ambros, 2003; Lai, 2003). Analyze the above-mentioned regulatory pathways by further consulting the data, and describing them according to the name (ID, category). Related to gene expression are Ribosome (03010, translation), Ribosomes biogenesis in eukaryotes (03008, translation), Ubiquitin mediated proteolysis (04120, folding, classification and degradation), and Aminoacyl-tRNA biosynthesis (00970, translation), Spliceosome (03040, transcription). Related to metabolism: Carbon metabolism (01200, global), Glycolysis/Gluconeogenesis (00010, carbohydrate metabolism), Arginine and proline metabolism (00330, amino acid metabolism), Citrate cycle (TCA cycle) (00020, carbohydrate metabolism), Biosynthesis of secondary metabolites (01110, global), Pyruvate metabolism (00620, carbohydrate metabolism), Lysine degradation (00310, amino acid metabolism), Tryptophan metabolism (00380, amino acid metabolism), Phenylalanine tyrosine and tryptophan biosynthesis (00400, amino acid metabolism), Metabolic pathways (01100, global), Carbon fixation in photosynthetic organisms (00710, energy metabolism). Related to defense is the Biosynthesis of secondary metabolites (01110).

The KEGG_Pathway that targets *Solanum tuberosum* and *Oryza sativa* is displayed according to the description of the pathway (false discovery rate of *Phytophthora infestans*, false discovery rate of *Magnaporthe oryzae*), Followed by Metabolic pathways (1.46E-07, 0.0322), Ribosome (2.16E-07, 1.30E-06), Carbon metabolism (1.26E-06, 1.11E-05), Ribosomal biogenesis in eukaryotes (1.41E-06, 6.25E-05), Glycolysis/Gluconeogenesis (9.15E-06, 0.00038), Biosynthesis of secondary metabolites (1.78E-05, 0.0049), Aminoacyl-tRNA biosynthesis (0.00099, 0.0031), Pyruvate metabolism (0.0055, 0.0062), Circadian rhythm-plant (0.0141, 0.00074), Pentose phosphate pathway (0.0211, 0.0031), Spliceosome (0.0282, 0.0291).

Among them, Ribosome (03010, translation), Ribosome biogenes in eukaryotes (03008, translation), Aminoacyl-tRNA biosynthesis (00970, translation), and Spliceosome (03040, translation) are related to gene expression. Related to metabolism: Metabolic pathway (01100, global), Carbon metabolism (01200, global), Glycolysis/Gluconeogenesis (00010, carbohydrate metabolism), Biosynthesis of secondary metabolites (01110, global), Pyruvate metabolism (00620, carbohydrate metabolism), Pentose phosphate pathway (00030, carbohydrate metabolism). Related to defense is the biosynthesis of secondary metabolites (01110). Circadian rhythm-Plants (04712) control many important physiological processes of plants, such as flowering and fruiting, growth, metabolism, and response to biotic and abiotic stresses (Carrington and Ambros, 2003; Lai, 2003).

For the KEGG_Pathway targeting *Solanum tuberosum* and *Solanum lycopersicum*, display according to the description of the pathway (false discovery rate of *Phytophthora infestans*, false discovery rate of *Botrytis cinerea*), followed by Metabolic pathway (1.46E-07, 1.60E-14), Ribosome (2.16E-07, 1.11E-05), Carbon metabolism (1.26E-06, 1.87E-12), Ribosomal biogenesis in eukaryotes (1.41E-06, 4.26E-06), RNA transport (2.38E-06, 0.00029), Glycolysis/Gluconeogenesis (9.15E-06, 0.0001), Glycine serine and threonine metabolism (1.61E-05, 0.0225), Biosynthesis of secondary metabolites (1.78 E-05, 5.66E-09), Biosynthesis of amino acids (0.00019, 1.11E-05), Purine metabolism (0.00029, 0.0352), Glyoxylate and dicarboxylate metabolism (0.00034, 0.0038), Aminoacyl-tRNA Biosynthesis (0.00099, 0.0011), Pyruvate metabolism (0.0055, 1.67E-11), Starch and sucrose metabolism (0.0115, 0.00068), Spliceosome (0.0282, 0.00057).

Among them are Ribosome (03010, translation), Ribosome biogenes in eukaryotes (03008, translation), RNA transport (03013, translation), Aminoacyl-tRNA biosynthesis (00970, translation), and Spliceosome (03040, transcription) related to gene expression. Related to metabolism: Metabolic pathways (01100, global), Carbon metabolism (01200, global), Glycolysis/Gluconeogenesis (00010, carbohydrate metabolism), Glycine serine and threonine metabolism (00260, amino acid metabolism), Biosynthesis of secondary metabolites (01110, global), Biosynthesis of amino acids (01230, global), Purine metabolism (00230, nucleotide metabolism), Glyoxylate and dicarboxylate metabolism (00630, carbohydrates metabolism), Pyruvate metabolism (00620, carbohydrate metabolism), Starch and sucrose metabolism (00500, carbohydrate metabolism). Related to defense is the Biosynthesis of secondary metabolites (01110).

**FIGURE 7:**
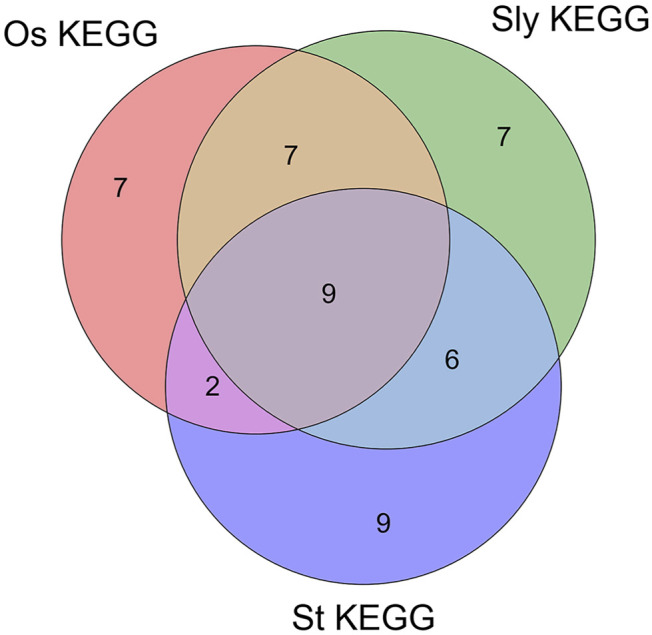
Venn diagram of three fungal sRNA targeting plant KEGG_pathway. In the figure, Os_KEGG is the KEGG_Pathway of *Magnaporthe oryzae* targeting *Oryza sativa*. Sly_KEGG is the KEGG_Pathway of *Botrytis cinerea* targeting *Solanum lycopersicum*. St_KEGG is the KEGG_Pathway of *Phytophthora infestans* targeting *Solanum tuberosum*.

**FIGURE 8:**
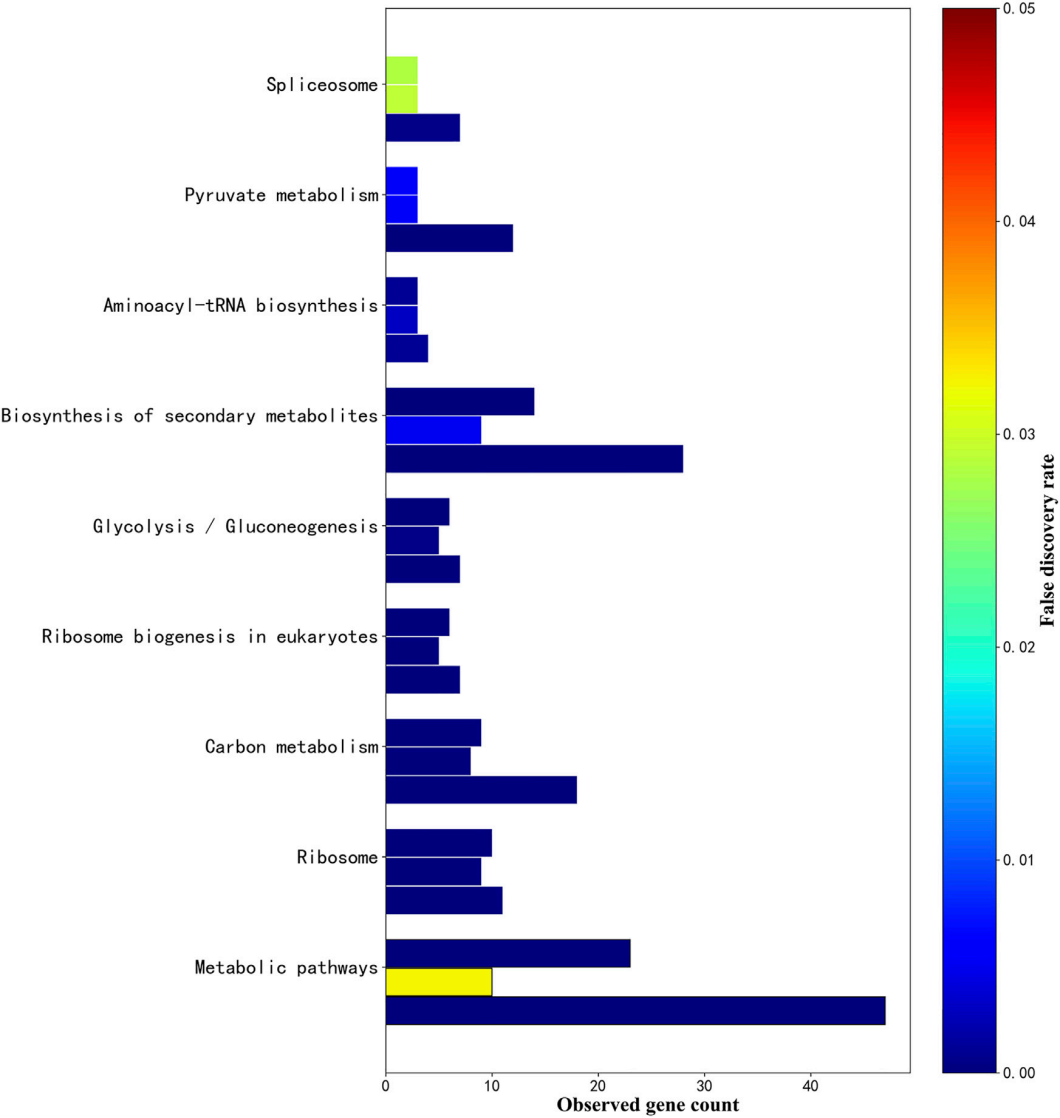
. KEGG_pathway parallel histogram of three fungal sRNA targeting. The figure above shows 9 KEGG_Pathways that co-exist in *Phytophthora infestans*, *Magnaporthe oryzae* and *Botrytis cinerea*. The upper bar graph is the KEGG_Pathway targeted by *Phytophthora infestans* sRNA, the bar in the middle is *Magnaporthe oryzae*, and the lower bar graph is the KEGG_Pathway targeted by *Botrytis cinerea* sRNA. The *x*-axis is the number of genes observed in the pathway. The *y*-axis in the figure is sorted in ascending order of the false discovery rate of *Phytophthora infestans*. The color of the false discovery rate is displayed in the right label.

There are 9 intersections of KEGG_Pathway that co-target the three plants, as shown in Figure 7. According to the description of the pathway (false discovery rate of *Phytophthora infestans*, false discovery rate of *Magnaporthe oryzae*, false discovery rate of *Botrytis cinerea*), the Metabolic pathways (1.46E-07, 0.0322, 1.60E-14), Ribosome (2.16E-07, 1.30E-06, 1.11E-05), Carbon metabolism (1.26E-06, 1.11E-05, 1.87E-12), Ribosome biogenesis in eukaryotes (1.41E-06, 6.25E-05, 4.26E-06), Glycolysis/Gluconeogenesis (9.15E-06, 0.00038, 0.0001), Biosynthesis of secondary metabolites (1.78E-05, 0.0049, 5.66E-09), Aminoacyl-tRNA biosynthesis (0.00099, 0.0031, 0.0011), Pyruvate metabolism (0.0055, 0.0062, 1.67E-11), Spliceosome (0.0282, 0.0291, 0.00057). The term description, observed gene count, and false discovery rate are shown in Figure 8.

Among the above-mentioned related to gene expression are Ribosome (03010, translation), Ribosome biogenesis in eukaryotes (03008, translation), Aminoacyl-tRNA biosynthesis (00970, translation), and Spliceosome (03040, transcription). Related to metabolism: Metabolic pathway (01100, global), Carbon metabolism (01200, global), Glycolysis/Gluconeogenesis (00010, carbohydrate metabolism), Biosynthesis of secondary metabolites (01110, global), Pyruvate metabolism (00620, carbohydrate metabolism). Related to defense is the Biosynthesis of secondary metabolites (01110).

### Comparison and Analysis of Models Based on Optimized Parameters

**FIGURE 9:**
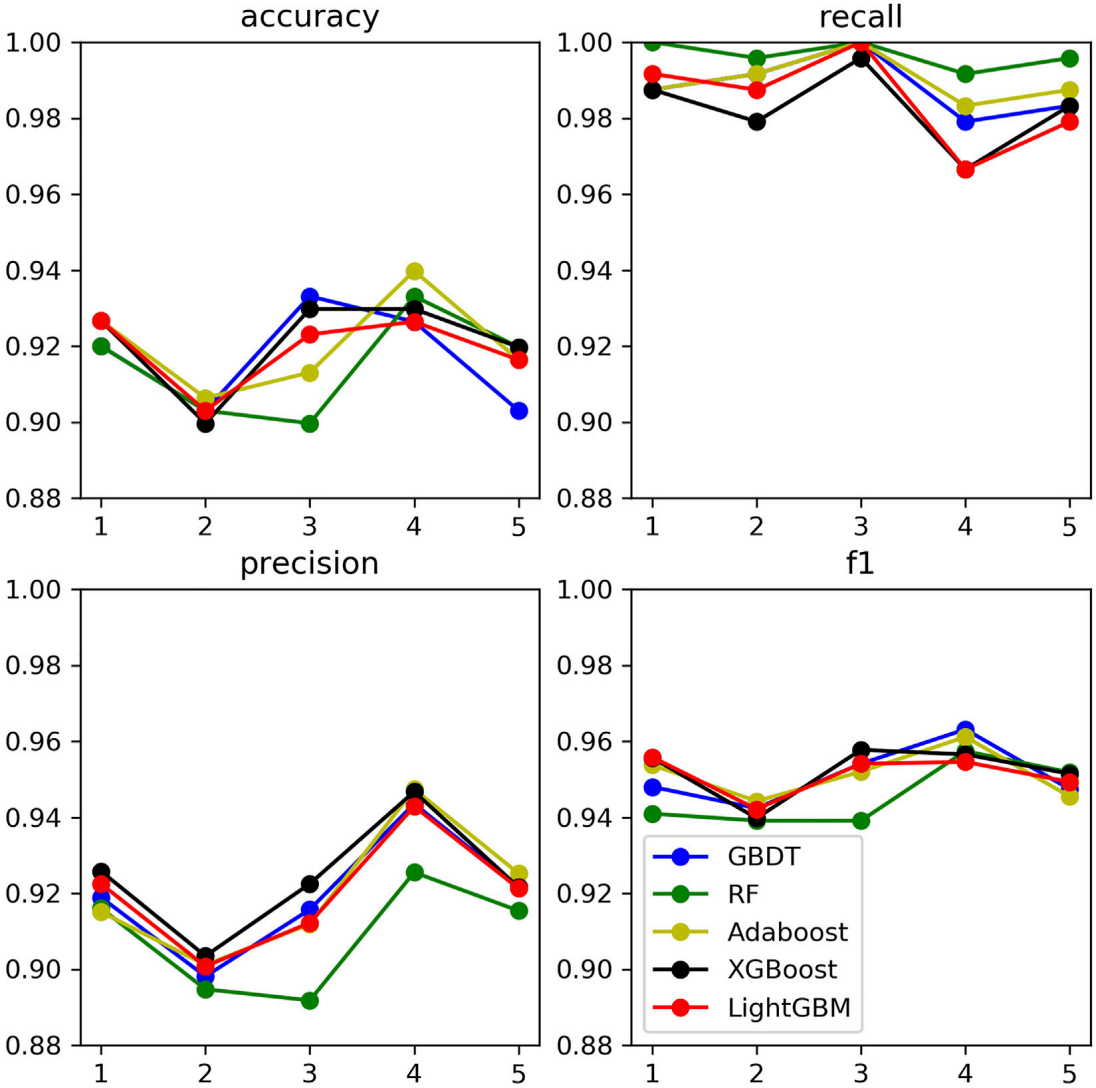
Evaluation of five models of *Botrytis cinerea*.

In order to compare the classification effects of the above three fungal data sets corresponding to the five ensemble learning models, this paper sets them to the parameters in Table 6. Then the five-fold cross-validation training sample set is used to calculate the four indicators. The results for *Botrytis cinerea* is shown in Figure 9. The corresponding results for *Magnaporthe oryzae* and *Phytophthora infestans* are shown in Supplementary Figures S3, S4 in the Supplementary file.

It can be found that the five ensemble learning models perform well under the four indicators. In addition, to prevent the machine learning model from comparing the predicted probability value and the threshold value to judge whether it is a key sRNA by the size. Using Receiver Operating Characteristic Curve to analyze the five integrated learning algorithms of three fungi separately, the area under the curve is the value of AUC. The ordinate of the curve is the recall rate, and the abscissa is the false positive rate (FRR). The specific calculation method is denoted in Eq. 8:

$$FPR=\frac{FP}{TN+FP}$$ (8)

**FIGURE 10:**
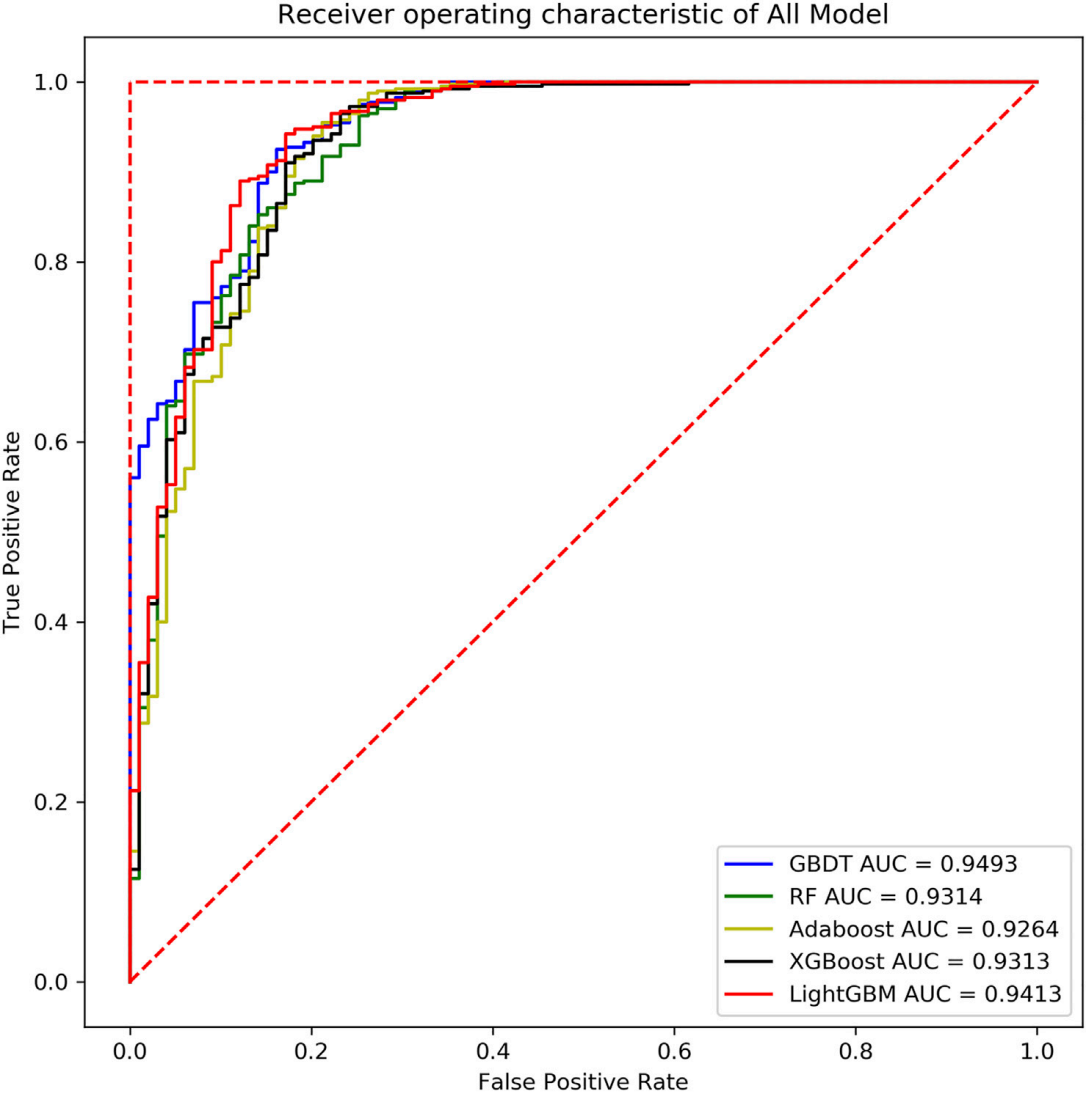
ROC curves of five models of *Botrytis cinerea*.

As shown below, Supplementary Figure S5 (in the supplementary file), Figure 10, and Supplementary Figure S6 (in the supplementary file) are the evaluation curve of the binary classification model constructed by the five ensemble learning models on the three fungal validation set samples, including 444 *Magnaporthe oryzae*, 499 *Botrytis cinerea* and 553 *Phytophthora infestans*.

Based on the analysis and summary of the above three figures, the following conclusions can be drawn: the area under the curve of the five ensemble learning models for the three fungi is relatively large, and the whole is smoother, and there is no under-fitting and over-fitting. In 2019, some researchers used the SVM model to predict the key sRNA of *Magnaporthe oryzae*, and the final model had an accuracy of 83% and an AUC of 0.85 (Hao et al., 2019). In 2020, some researchers used the Random Forest model to predict the key sRNA of *Phytophthora infestans*, and the final model had an accuracy of 85.23% and an AUC of 0.9169 (Liu et al., 2020). Some researchers used five models of Light Gradient Boosting Machine, Random Forest, KNN, Classification And Regression Tree, and SVM to predict the key sRNAs of *Phytophthora infestans* pathogenicity, and compared them with multiple indicators, and found that Random Forest was significantly better than other classification models in all indicators. Using KNN, SVM, Naive Bayes, Decision Tree, Random Forest and Adaboost six models to predict the key sRNA of *Magnaporthe oryzae* pathogenicity, it is found that Adaboost has a better classification effect, SVM and Random Forest have Over-fitting phenomenon, KNN, Naive Bayes (NB), Decision Tree prediction effect is poor (Zhang et al., 2020). In this paper, it can be found that GBDT, XGBoost, and Light GBM perform better than Random Forest and Adaboost. The accuracy and AUC statistical results of the training set and validation set are shown in Table 7. Since *Magnaporthe oryzae* has fewer key sRNAs, its accuracy, precision, and AUC value are lower than those of the other two fungi. Therefore, this paper selects the XGBoost model with the highest AUC value on *Magnaporthe oryzae* and higher AUC values in the other two fungi. The model performs well under other indicators as the final model.

**TABLE 7:** The accuracy and AUC statistics of the three models of the three fungi.

Model	GBDT			XGBoost			LightGBM		
Training set accuracy	0.88	0.96	0.96	0.88	0.96	0.97	0.87	0.95	0.97
Validation set accuracy	0.84	0.93	0.88	0.85	0.93	0.89	0.85	0.93	0.89
Training set AUC	0.92	0.99	0.99	0.93	0.99	0.99	0.92	0.99	0.99
Validation set AUC	0.84	0.95	0.90	0.86	0.93	0.90	0.85	0.94	0.91

## Discussion

### Limitations of sRNA Sequence

This article only analyzes the up-regulated sequences with apparent changes in fungal sRNA expression and does not involve the study of down-regulated sequences and differentially expressed sequences of plant sRNA. The may be related to plant defense and resistance to fungi (Fei et al., 2016; Zanini et al., 2019), and its role needs to be further explored. On the other hand, because this article only studies the sRNA data before and after the infection of the three fungi from the database, and only the data 72 h after infection. Because this is a newer direction, there are insufficient data. In response to this problem, the class method can be used, that is, using the existing homologous fungal sRNA or sRNA after fungal infection of homologous plants, if they have common fungal sRNA and plant mRNA, their targeting has more similarity. In addition, studies have shown that spraying artificially synthesized sRNAs targeting pathogen virulence-related genes on plants can inhibit the infection and growth of fungi (Koch et al., 2016; Wang and Jin, 2017; Zhu et al., 2019). Despite the high cost of sRNA synthesis and short shelf life, the future use of sRNAs as biopesticides is promising compared to the time required to breed pathogen-resistant crops to obtain stable transgenic lines (Wang et al., 2016). Therefore, in the near future, there will be more and more relevant data. In the case of sufficient data in the future, based on the research process of this article, we can study more kinds of fungal sRNA transboundary regulation of plants and plant sRNA transboundary regulation of fungi, explore the interaction mechanism between fungi and plants in order to find out more comprehensive commonality and difference. This will bring new ideas for plant prevention and control of fungal diseases, and provide a more comprehensive theoretical basis for formulating persistent and broad-spectrum methods for plant prevention and control of fungi.

### Discover the Significance of Biological Processes and Regulatory Pathways

In this paper, the three key sRNA sequences are respectively targeted to the corresponding plant mRNA/CDS/transcripts, and then mapped to the corresponding genes for functional enrichment analysis. The large number of nodes makes screening out the core nodes for the final functional enrichment analysis necessary. The study found that three fungi target multiple GO and multiple KEGG_Pathway. The commonality analysis of the three fungal targets found that multiple GO and multiple KEGG_Pathway coexisted, and the KEGG_Pathway was further reviewed and analyzed. This article analyzes the co-existing KEGG_Pathway by further consulting data. The results of this paper show that fungal sRNAs have certain commonalities in transboundary regulation of plants, and the key sRNA sequences mined can participate in the regulation of plant gene expression and metabolism, etc., which affect plant growth, development, reproduction, and response to external environments. In addition, this article only analyzes the fungal cross-species regulation of plants from the data level and the theoretical level, but there are few related studies. Therefore, whether the research is entirely correct or not needs to be verified by biological experiments. However, many researchers have found through biological experiments that fungal differentially expressed sRNAs are involved in the necrotrophic infection phase, including gene expression in metabolism, translation-related and defense responses (Zanini et al., 2019; Hunt et al., 2019; Derbyshire et al., 2019), which is consistent with the conclusions of this paper. This laid a theoretical foundation for preventing and controlling fungal diseases and opened up new research directions.

### The Universality of the Model

Because organisms are divided into large to small categories in kingdoms, phylums, classes, woods, families, genera, and species. Among the three plants, *Solanum lycopersicum* and *Solanum tuberosum* belong to the same genus (Solanum), while *Oryza sativa* is very different from *Solanum lycopersicum* and *Solanum tuberosum* and belongs to the same phylum (plant phylum). Among the three fungi, *Magnaporthe oryzae*, *Botrytis cinerea*, and *Phytophthora infestans* belong to the same kingdom, and they are three completely different phyla. The larger the category gap, the larger the genetic gap. This study used three completely different fungi to infect three plants with huge differences, and the XGBoost model performed very well, so this model can not only be used to predict the key sRNAs of the three fungi that regulate the three plants across borders in this article. The model can also be used to predict the key sRNAs of other fungi that regulate plants across borders, and the predicted classification results have reference value. In addition, among a variety of machine learning models, there are many models that are suitable for binary classification prediction of samples. Some studies have shown that the fusion of models with larger differences has a better classification effect (Zhang et al., 2020). Researchers can also identify important features of pathogenic key sRNAs, and mine the commonalities of pathogenic sRNA characteristics.

## Data Availability

The original contributions presented in the study are included in the article/Supplementary Material, further inquiries can be directed to the corresponding author.
