# Supplementary material for: Exploring the Common Mechanism of Fungal sRNA Transboundary Regulation of Plants Based on Ensemble Learning Methods
Source: Front Genet. 2022 Feb 11;13:816478. doi: 10.3389/fgene.2022.816478 (PMC8873571; doi:10.3389/fgene.2022.816478)

Supplementary Material

# Supplementary Tables

Table S1. *Magnaporthe oryzae* core node and its degree.

| node | degree |
| --- | --- |
| OsJ_17309 | 31 |
| OsJ_15986 | 27 |
| PES | 27 |
| OS07T0603200-01 | 26 |
| OsJ_23326 | 20 |
| OsJ_12950 | 20 |
| OsJ_32023 | 20 |
| OsJ_35136 | 20 |
| OsJ_26117 | 19 |
| OsJ_25933 | 18 |
| OsJ_10517 | 17 |
| OS09T0551300-02 | 17 |
| OS05T0535800-00 | 16 |
| OsJ_34468 | 15 |
| OS02T0724600-01 | 15 |
| RPS4 | 14 |
| OsJ_24764 | 14 |
| OS11T0222200-01 | 14 |
| OsJ_19902 | 13 |
| OsJ_09668 | 13 |
| OsRPL14 | 13 |
| OS02T0146700-02 | 13 |
| OS02T0795900-01 | 13 |
| OsJ_02628 | 12 |
| OS02T0710900-01 | 11 |
| NIA1 | 11 |
| OS03T0722600-01 | 11 |
| H2B.10 | 10 |
| OsJ_12779 | 10 |
| H2B.5 | 9 |
| H2B.6 | 9 |
| SRP19 | 9 |
| OsJ_001872 | 9 |
| H2B.11 | 9 |
| H2B.9 | 9 |
| H2B.7 | 9 |
| PRMT1 | 9 |
| OsJ_04005 | 9 |
| OS03T0406200-01 | 9 |
| OS03T0339100-01 | 8 |
| CDKA-2 | 8 |
| OS12T0277500-01 | 8 |
| JMJ705 | 8 |
| OsJ_19507 | 8 |
| AGO1C | 8 |
| OsJ_05902 | 8 |
| RBOHB | 8 |
| OS07T0508000-01 | 7 |
| OsJ_06469 | 7 |
| OsJ_28120 | 7 |
| OsJ_06853 | 7 |
| PHYC | 7 |
| OsJ_31649 | 7 |
| OsJ_11040 | 7 |
| OS07T0659800-01 | 7 |
| OS01T0123700-00 | 7 |
| OsJ_31005 | 7 |
| OsJ_21204 | 7 |
| OS06T0343100-01 | 7 |
| OS03T0765000-01 | 7 |
| OS03T0298700-01 | 7 |
| OS02T0285800-01 | 7 |
| OsJ_25175 | 7 |
| PPS | 6 |
| GI | 6 |
| RSZ21A | 6 |
| OsJ_31437 | 6 |
| OS03T0257600-01 | 6 |
| OsJ_20480 | 6 |
| OsJ_29674 | 6 |
| OsJ_28086 | 6 |
| OS12T0616900-01 | 6 |
| OsJ_30298 | 6 |
| OsCRY2 | 5 |
| OsJ_03057 | 5 |
| OsJ_09152 | 5 |
| OsJ_33746 | 5 |
| OsJ_06017 | 5 |
| OS06T0548000-01 | 5 |
| OS09T0482000-01 | 5 |
| OS03T0111800-01 | 5 |
| JAR1 | 5 |
| GH3.4 | 5 |
| OsJ_07584 | 5 |
| OS01T0761500-01 | 5 |
| OS10T0370700-01 | 5 |
| OS07T0603800-01 | 5 |
| OS08T0425700-01 | 5 |
| OS09T0440300-01 | 5 |
| OsJ_07404 | 5 |
| OS04T0390000-02 | 5 |
| OS08T0359000-01 | 5 |
| ATG4B | 5 |
| CDKD-1 | 5 |
| OsJ_02543 | 5 |
| OsJ_19625 | 5 |

Table S2. *Botrytis cinerea* core node and its degree.

| node | degree |
| --- | --- |
| RPE1 | 49 |
| 101243636 | 37 |
| 101256673 | 37 |
| 101264867 | 37 |
| 101247532 | 37 |
| 101249838 | 34 |
| 101257282 | 34 |
| 101250574 | 33 |
| 101263844 | 31 |
| 101257307 | 30 |
| 101258943 | 30 |
| 101245558 | 29 |
| 101254120 | 28 |
| 101247310 | 27 |
| 101248302 | 27 |
| 101253805 | 26 |
| 101263645 | 24 |
| 101265477 | 24 |
| 101244293 | 24 |
| 101245686 | 24 |
| 101249025 | 23 |
| 101243936 | 23 |
| 101259018 | 23 |
| 101255780 | 23 |
| 101261833 | 23 |
| PRO2 | 23 |
| 101246051 | 22 |
| 101258729 | 22 |
| 101249010 | 22 |
| 101253561 | 22 |
| Solyc11g062010.1.1 | 22 |
| 101258079 | 21 |
| Solyc05g023710.2.1 | 20 |
| 101254504 | 20 |
| 101251229 | 20 |
| 101263722 | 20 |
| 101259798 | 19 |
| 101243864 | 19 |
| chMDH | 19 |
| mMDH | 19 |
| 101252799 | 19 |
| Solyc03g112530.2.1 | 19 |
| 101247472 | 19 |
| 101250741 | 19 |
| 101250733 | 18 |
| Solyc01g081050.2.1 | 18 |
| 101251184 | 18 |
| Solyc01g086800.2.1 | 17 |
| 101246924 | 17 |
| 101250600 | 17 |
| 101259587 | 17 |
| 101251648 | 16 |
| 101249527 | 16 |
| Solyc12g038200.1.1 | 16 |
| 101250511 | 16 |
| 101248917 | 16 |
| HSC80 | 16 |
| 101257775 | 16 |
| 101245956 | 16 |
| 101246256 | 16 |
| Solyc11g042870.1.1 | 16 |
| 101266598 | 16 |
| MET | 15 |
| 101268630 | 15 |
| 101264020 | 15 |
| DHX35 | 15 |
| 101265681 | 15 |
| 101267346 | 15 |
| 101262595 | 15 |
| Solyc06g062500.2.1 | 15 |
| 101261454 | 15 |
| 101259902 | 15 |
| Solyc10g006770.2.1 | 14 |
| 101247309 | 14 |
| 101247897 | 14 |
| 101258879 | 14 |
| 101247869 | 14 |
| Solyc01g080840.2.1 | 14 |
| Solyc07g021380.1.1 | 14 |
| MDN1 | 14 |
| Solyc07g039330.2.1 | 13 |
| Solyc11g020290.1.1 | 13 |
| 101251072 | 13 |
| 101260947 | 13 |
| 101245359 | 13 |
| 101245649 | 13 |
| 101259035 | 13 |
| Solyc05g045830.1.1 | 13 |
| Solyc03g082920.2.1 | 13 |
| Solyc06g052050.2.1 | 13 |
| MDC | 13 |
| LOC543812 | 13 |
| LOC543968 | 12 |
| 101252849 | 12 |
| 101252346 | 12 |
| wee1 | 12 |
| Solyc12g013880.1.1 | 12 |
| 101254008 | 12 |
| Solyc01g110990.2.1 | 12 |
| Solyc11g072520.1.1 | 12 |
| Solyc01g106770.2.1 | 12 |
| Solyc12g035360.1.1 | 12 |
| Solyc09g010460.2.1 | 12 |
| HXK4 | 12 |
| 101252851 | 11 |
| 101261453 | 11 |
| Solyc02g085390.2.1 | 11 |
| Solyc12g005080.1.1 | 11 |
| 101254921 | 11 |
| Solyc03g033430.1.1 | 11 |
| Solyc01g073970.2.1 | 11 |
| Solyc01g074000.2.1 | 11 |
| Solyc01g086820.2.1 | 11 |
| 101243735 | 10 |
| Solyc05g026510.2.1 | 10 |
| 101268334 | 10 |
| Solyc06g060350.2.1 | 10 |
| Solyc01g099220.2.1 | 10 |
| 101249170 | 10 |
| 101266713 | 10 |
| 101261919 | 10 |
| Solyc04g057990.2.1 | 10 |
| 101249760 | 10 |
| SSADH | 10 |
| 101249217 | 10 |
| 101266536 | 10 |
| 101248407 | 10 |
| ppc1 | 10 |
| 101255240 | 10 |
| 101259903 | 10 |
| 101264146 | 10 |
| 101248519 | 10 |
| 101264112 | 10 |
| 101253199 | 10 |
| 101257856 | 10 |
| Solyc03g019960.2.1 | 9 |
| Solyc02g032550.1.1 | 9 |
| 101267921 | 9 |
| 101259128 | 9 |
| Solyc04g016010.2.1 | 9 |
| RAN1 | 9 |
| 101252961 | 9 |
| TPG6 | 9 |
| mpka1;1 | 9 |
| Solyc07g065630.2.1 | 9 |
| Solyc04g009140.2.1 | 9 |
| 101266363 | 9 |
| 101260717 | 9 |
| 101257857 | 9 |
| 101251299 | 9 |
| Solyc06g005080.2.1 | 9 |
| 101263337 | 9 |
| Solyc08g067040.2.1 | 9 |
| 101263950 | 9 |
| 101250549 | 9 |
| CYP51 | 9 |
| AGO6 | 9 |
| 101268268 | 9 |
| Solyc04g012170.2.1 | 9 |
| 100316881 | 8 |
| AGP-S1 | 8 |
| 101245879 | 8 |
| 101260712 | 8 |
| 101267413 | 8 |
| LOC543929 | 8 |
| 101265278 | 8 |
| 101244224 | 8 |
| Solyc01g109360.2.1 | 8 |
| 101250390 | 8 |
| Solyc03g122310.2.1 | 8 |
| 101265811 | 8 |
| 101244858 | 8 |
| 101245889 | 8 |
| 101257065 | 8 |
| GATC | 8 |
| 101259924 | 8 |
| 101247946 | 8 |
| 101260021 | 8 |
| 101251928 | 8 |
| Solyc01g058380.1.1 | 8 |
| 101263943 | 8 |
| 101249338 | 8 |
| Solyc08g079110.2.1 | 8 |
| Solyc04g072390.2.1 | 8 |
| 101252510 | 8 |
| 101260365 | 8 |
| Solyc04g076620.2.1 | 8 |
| 101258649 | 8 |
| 101258308 | 8 |
| 101249619 | 8 |
| 101259137 | 8 |
| 101263336 | 8 |
| SUS4 | 8 |
| 101266596 | 8 |
| 101267641 | 8 |
| Solyc04g077440.2.1 | 8 |
| 101268335 | 8 |

Table S3. *Phytophthora infestans* core node and its degree.

| node | degree |
| --- | --- |
| 102578407 | 37 |
| PGSC0003DMT400079915 | 34 |
| 102603701 | 34 |
| 102593560 | 27 |
| 102587196 | 27 |
| 102582667 | 26 |
| PGSC0003DMT400059174 | 25 |
| 102587574 | 25 |
| PGSC0003DMT400066770 | 25 |
| PGSC0003DMT400024801 | 25 |
| PGSC0003DMT400034496 | 25 |
| 102603891 | 25 |
| PGSC0003DMT400002872 | 24 |
| 102579491 | 24 |
| 102581875 | 24 |
| 102605472 | 23 |
| PGSC0003DMT400021952 | 23 |
| 102595553 | 23 |
| 102603390 | 23 |
| 102604659 | 22 |
| 102605043 | 22 |
| PGSC0003DMT400044808 | 21 |
| 102603859 | 20 |
| 102586674 | 19 |
| 102590512 | 19 |
| 102580337 | 18 |
| PGSC0003DMT400005427 | 17 |
| 102580154 | 17 |
| 102585048 | 16 |
| 102587109 | 16 |
| 102585784 | 16 |
| 102590182 | 16 |
| 102603797 | 16 |
| 102587684 | 14 |
| 102606382 | 13 |
| 102597065 | 12 |
| PGSC0003DMT400016911 | 12 |
| 102604222 | 11 |
| 102594613 | 11 |
| 102586442 | 11 |
| 102604824 | 11 |
| 102596272 | 11 |
| 102601905 | 11 |
| 102605977 | 11 |
| 102582645 | 11 |
| 102588992 | 11 |
| 102585342 | 11 |
| PGSC0003DMT400006585 | 11 |
| PGSC0003DMT400083452 | 11 |
| 102600021 | 10 |
| 102604688 | 10 |
| 102604773 | 10 |
| 102585761 | 10 |
| 102600125 | 10 |
| 102606212 | 10 |
| 102584723 | 10 |
| 102577433 | 9 |
| 102592969 | 9 |
| 102589798 | 9 |
| PGSC0003DMT400078197 | 9 |
| PGSC0003DMT400053661 | 9 |
| PGSC0003DMT400021038 | 9 |
| 102588127 | 9 |
| 102598532 | 9 |
| PGSC0003DMT400021697 | 9 |
| PGSC0003DMT400021699 | 9 |
| 102604583 | 8 |
| PGSC0003DMT400063605 | 8 |
| 102580238 | 7 |
| 102578396 | 7 |
| 102591223 | 7 |
| 102578859 | 7 |
| 102590431 | 7 |
| 102604163 | 7 |
| PGSC0003DMT400073084 | 7 |
| 102593580 | 7 |
| 102596937 | 7 |
| PGSC0003DMT400072632 | 7 |
| InvGF | 7 |
| 102592163 | 6 |
| PGSC0003DMT400007721 | 6 |
| 102579136 | 6 |
| 102605580 | 6 |
| 102596668 | 6 |
| PGSC0003DMT400028915 | 6 |
| 102595124 | 6 |
| 102582524 | 6 |
| PGSC0003DMT400002595 | 6 |
| 107058768 | 6 |
| 102594988 | 6 |
| 102584902 | 6 |
| PGSC0003DMT400079885 | 6 |
| Pain-1 | 6 |
| 102598451 | 6 |
| 102597582 | 6 |
| 102599296 | 5 |
| 102585058 | 5 |
| 102599270 | 5 |
| 102580017 | 5 |
| CHS1B | 5 |
| 102582206 | 5 |
| 102583232 | 5 |
| 102590502 | 5 |
| 102582317 | 5 |
| 102582462 | 5 |
| PGSC0003DMT400081297 | 5 |
| 102601840 | 5 |
| 102601663 | 5 |
| 102598888 | 5 |
| 102604947 | 5 |
| 102590563 | 5 |
| PGSC0003DMT400024220 | 5 |

Table S4. Information of the Molecular Function (Gene Ontology) targeted by *Magnaporthe oryzae*.

| #term ID | term description | observed gene count | background gene count | strength | false discovery rate | matching proteins in your network (IDs) | matching proteins in your network (labels) |
| --- | --- | --- | --- | --- | --- | --- | --- |
| GO:0046982 | protein heterodimerization activity | 7 | 33 | 1.9 | 1.71E-09 | 4530.OS01T0152300-00 4530.OS01T0152900-00 4530.OS01T0153100-00 4530.OS01T0153300-00 4530.OS01T0839500-00 4530.OS03T0279200-01 4530.OS05T0574300-00 | H2B.10 H2B.7 H2B.6 H2B.5 H2B.11 OsJ_001872 H2B.9 |
| GO:0003676 | nucleic acid binding | 16 | 828 | 0.86 | 4.12E-08 | 4530.OS01T0152300-00 4530.OS01T0152900-00 4530.OS01T0153100-00 4530.OS01T0153300-00 4530.OS01T0618400-01 4530.OS01T0839500-00 4530.OS01T0907400-01 4530.OS02T0105900-01 4530.OS02T0795900-01 4530.OS02T0831600-01 4530.OS03T0279200-01 4530.OS03T0406200-01 4530.OS03T0699200-01 4530.OS05T0574300-00 4530.OS06T0187900-02 4530.OS06T0342100-01 | H2B.10 H2B.7 H2B.6 H2B.5 OsJ_02628 H2B.11 JMJ705 RPS4 OS02T0795900-01 AGO1C OsJ_001872 OS03T0406200-01 PES H2B.9 RSZ21A SRP19 |
| GO:0097159 | organic cyclic compound binding | 21 | 1523 | 0.71 | 4.12E-08 | 4530.OS01T0152300-00 4530.OS01T0152900-00 4530.OS01T0153100-00 4530.OS01T0153300-00 4530.OS01T0618400-01 4530.OS01T0839500-00 4530.OS01T0907400-01 4530.OS02T0105900-01 4530.OS02T0123100-01 4530.OS02T0506500-04 4530.OS02T0795900-01 4530.OS02T0831600-01 4530.OS03T0279200-01 4530.OS03T0406200-01 4530.OS03T0699200-01 4530.OS05T0392300-01 4530.OS05T0574300-00 4530.OS06T0187900-02 4530.OS06T0342100-01 4530.OS08T0468100-01 4530.OS09T0556500-01 | H2B.10 H2B.7 H2B.6 H2B.5 OsJ_02628 H2B.11 JMJ705 RPS4 CDKA-2 OsJ_06853 OS02T0795900-01 AGO1C OsJ_001872 OS03T0406200-01 PES CDKD-1 H2B.9 RSZ21A SRP19 NIA1 OsJ_30298 |
| GO:1901363 | heterocyclic compound binding | 21 | 1523 | 0.71 | 4.12E-08 | 4530.OS01T0152300-00 4530.OS01T0152900-00 4530.OS01T0153100-00 4530.OS01T0153300-00 4530.OS01T0618400-01 4530.OS01T0839500-00 4530.OS01T0907400-01 4530.OS02T0105900-01 4530.OS02T0123100-01 4530.OS02T0506500-04 4530.OS02T0795900-01 4530.OS02T0831600-01 4530.OS03T0279200-01 4530.OS03T0406200-01 4530.OS03T0699200-01 4530.OS05T0392300-01 4530.OS05T0574300-00 4530.OS06T0187900-02 4530.OS06T0342100-01 4530.OS08T0468100-01 4530.OS09T0556500-01 | H2B.10 H2B.7 H2B.6 H2B.5 OsJ_02628 H2B.11 JMJ705 RPS4 CDKA-2 OsJ_06853 OS02T0795900-01 AGO1C OsJ_001872 OS03T0406200-01 PES CDKD-1 H2B.9 RSZ21A SRP19 NIA1 OsJ_30298 |
| GO:0046983 | protein dimerization activity | 8 | 124 | 1.38 | 6.90E-08 | 4530.OS01T0152300-00 4530.OS01T0152900-00 4530.OS01T0153100-00 4530.OS01T0153300-00 4530.OS01T0839500-00 4530.OS03T0279200-01 4530.OS03T0752100-01 4530.OS05T0574300-00 | H2B.10 H2B.7 H2B.6 H2B.5 H2B.11 OsJ_001872 PHYC H2B.9 |
| GO:0005488 | binding | 24 | 2265 | 0.6 | 1.18E-07 | 4530.OS01T0152300-00 4530.OS01T0152900-00 4530.OS01T0153100-00 4530.OS01T0153300-00 4530.OS01T0360200-01 4530.OS01T0618400-01 4530.OS01T0839500-00 4530.OS01T0907400-01 4530.OS02T0105900-01 4530.OS02T0123100-01 4530.OS02T0506500-04 4530.OS02T0795900-01 4530.OS02T0831600-01 4530.OS03T0279200-01 4530.OS03T0406200-01 4530.OS03T0699200-01 4530.OS03T0752100-01 4530.OS05T0392300-01 4530.OS05T0574300-00 4530.OS05T0586200-01 4530.OS06T0187900-02 4530.OS06T0342100-01 4530.OS08T0468100-01 4530.OS09T0556500-01 | H2B.10 H2B.7 H2B.6 H2B.5 RBOHB OsJ_02628 H2B.11 JMJ705 RPS4 CDKA-2 OsJ_06853 OS02T0795900-01 AGO1C OsJ_001872 OS03T0406200-01 PES PHYC CDKD-1 H2B.9 JAR1 RSZ21A SRP19 NIA1 OsJ_30298 |
| GO:0005515 | protein binding | 10 | 366 | 1.01 | 1.30E-06 | 4530.OS01T0152300-00 4530.OS01T0152900-00 4530.OS01T0153100-00 4530.OS01T0153300-00 4530.OS01T0360200-01 4530.OS01T0839500-00 4530.OS03T0279200-01 4530.OS03T0752100-01 4530.OS05T0574300-00 4530.OS05T0586200-01 | H2B.10 H2B.7 H2B.6 H2B.5 RBOHB H2B.11 OsJ_001872 PHYC H2B.9 JAR1 |
| GO:0003824 | catalytic activity | 17 | 1818 | 0.54 | 0.00011 | 4530.OS01T0360200-01 4530.OS01T0618400-01 4530.OS01T0907400-01 4530.OS02T0123100-01 4530.OS02T0506500-04 4530.OS02T0795900-01 4530.OS03T0752100-01 4530.OS03T0776000-01 4530.OS04T0682000-01 4530.OS05T0392300-01 4530.OS05T0500900-01 4530.OS05T0586200-01 4530.OS08T0468100-01 4530.OS08T0536000-01 4530.OS09T0359800-01 4530.OS09T0556500-01 4530.OS12T0616900-01 | RBOHB OsJ_02628 JMJ705 CDKA-2 OsJ_06853 OS02T0795900-01 PHYC OsJ_12779 ATG4B CDKD-1 GH3.4 JAR1 NIA1 OsJ_28086 PRMT1 OsJ_30298 OS12T0616900-01 |
| GO:0003723 | RNA binding | 6 | 218 | 1.01 | 0.00045 | 4530.OS01T0618400-01 4530.OS02T0105900-01 4530.OS02T0795900-01 4530.OS03T0406200-01 4530.OS03T0699200-01 4530.OS06T0342100-01 | OsJ_02628 RPS4 OS02T0795900-01 OS03T0406200-01 PES SRP19 |
| GO:0016874 | ligase activity | 4 | 65 | 1.36 | 0.00045 | 4530.OS02T0506500-04 4530.OS05T0500900-01 4530.OS05T0586200-01 4530.OS09T0556500-01 | OsJ_06853 GH3.4 JAR1 OsJ_30298 |
| GO:0060089 | molecular transducer activity | 3 | 40 | 1.45 | 0.0024 | 4530.OS02T0123100-01 4530.OS03T0752100-01 4530.OS05T0392300-01 | CDKA-2 PHYC CDKD-1 |
| GO:0004739 | pyruvate dehydrogenase (acetyl-transferring) activity | 2 | 7 | 2.03 | 0.0026 | 4530.OS08T0536000-01 4530.OS12T0616900-01 | OsJ_28086 OS12T0616900-01 |
| GO:0004693 | cyclin-dependent protein serine/threonine kinase activity | 2 | 15 | 1.69 | 0.0077 | 4530.OS02T0123100-01 4530.OS05T0392300-01 | CDKA-2 CDKD-1 |
| GO:0008353 | RNA polymerase II CTD heptapeptide repeat kinase activity | 2 | 15 | 1.69 | 0.0077 | 4530.OS02T0123100-01 4530.OS05T0392300-01 | CDKA-2 CDKD-1 |
| GO:0140096 | catalytic activity acting on a protein | 6 | 420 | 0.72 | 0.0077 | 4530.OS01T0907400-01 4530.OS02T0123100-01 4530.OS03T0752100-01 4530.OS04T0682000-01 4530.OS05T0392300-01 4530.OS09T0359800-01 | JMJ705 CDKA-2 PHYC ATG4B CDKD-1 PRMT1 |
| GO:0003677 | DNA binding | 7 | 614 | 0.63 | 0.0094 | 4530.OS01T0152300-00 4530.OS01T0152900-00 4530.OS01T0153100-00 4530.OS01T0153300-00 4530.OS01T0839500-00 4530.OS03T0279200-01 4530.OS05T0574300-00 | H2B.10 H2B.7 H2B.6 H2B.5 H2B.11 OsJ_001872 H2B.9 |
| GO:0140098 | catalytic activity acting on RNA | 3 | 100 | 1.05 | 0.016 | 4530.OS01T0618400-01 4530.OS02T0795900-01 4530.OS09T0556500-01 | OsJ_02628 OS02T0795900-01 OsJ_30298 |
| GO:0005524 | ATP binding | 6 | 548 | 0.61 | 0.0215 | 4530.OS01T0618400-01 4530.OS02T0123100-01 4530.OS02T0506500-04 4530.OS02T0795900-01 4530.OS05T0392300-01 4530.OS09T0556500-01 | OsJ_02628 CDKA-2 OsJ_06853 OS02T0795900-01 CDKD-1 OsJ_30298 |
| GO:0016491 | oxidoreductase activity | 5 | 401 | 0.67 | 0.0238 | 4530.OS01T0360200-01 4530.OS01T0907400-01 4530.OS08T0468100-01 4530.OS08T0536000-01 4530.OS12T0616900-01 | RBOHB JMJ705 NIA1 OsJ_28086 OS12T0616900-01 |
| GO:0008144 | drug binding | 6 | 598 | 0.57 | 0.0273 | 4530.OS01T0618400-01 4530.OS02T0123100-01 4530.OS02T0506500-04 4530.OS02T0795900-01 4530.OS05T0392300-01 4530.OS09T0556500-01 | OsJ_02628 CDKA-2 OsJ_06853 OS02T0795900-01 CDKD-1 OsJ_30298 |
| GO:0043167 | ion binding | 10 | 1469 | 0.4 | 0.0277 | 4530.OS01T0360200-01 4530.OS01T0618400-01 4530.OS01T0907400-01 4530.OS02T0123100-01 4530.OS02T0506500-04 4530.OS02T0795900-01 4530.OS05T0392300-01 4530.OS06T0187900-02 4530.OS08T0468100-01 4530.OS09T0556500-01 | RBOHB OsJ_02628 JMJ705 CDKA-2 OsJ_06853 OS02T0795900-01 CDKD-1 RSZ21A NIA1 OsJ_30298 |
| GO:0016772 | transferase activity transferring phosphorus-containing groups | 4 | 296 | 0.7 | 0.0328 | 4530.OS02T0123100-01 4530.OS03T0752100-01 4530.OS05T0392300-01 4530.OS05T0586200-01 | CDKA-2 PHYC CDKD-1 JAR1 |
| GO:0003724 | RNA helicase activity | 2 | 58 | 1.11 | 0.0413 | 4530.OS01T0618400-01 4530.OS02T0795900-01 | OsJ_02628 OS02T0795900-01 |
| GO:0008186 | RNA-dependent ATPase activity | 2 | 58 | 1.11 | 0.0413 | 4530.OS01T0618400-01 4530.OS02T0795900-01 | OsJ_02628 OS02T0795900-01 |
| GO:0004672 | protein kinase activity | 3 | 187 | 0.78 | 0.0476 | 4530.OS02T0123100-01 4530.OS03T0752100-01 4530.OS05T0392300-01 | CDKA-2 PHYC CDKD-1 |

Table S5. Information of the KEGG_Pathway targeted by *Magnaporthe oryzae*.

| #term ID | term description | observed gene count | background gene count | strength | false discovery rate | matching proteins in your network (IDs) | matching proteins in your network (labels) |
| --- | --- | --- | --- | --- | --- | --- | --- |
| map03010 | Ribosome | 9 | 240 | 1.14 | 1.30E-06 | 4530.OS02T0105900-01 4530.OS02T0622300-00 4530.OS03T0182600-02 4530.OS03T0796501-00 4530.OS05T0535800-00 4530.OS06T0115500-01 4530.OS07T0180900-01 4530.OS11T0135400-00 4530.OS12T0133050-00 | RPS4 OsRPL14 OsJ_09668 OsJ_12950 OS05T0535800-00 OsJ_19902 OsJ_23326 OsJ_25933 OsJ_35136 |
| map01200 | Carbon metabolism | 8 | 245 | 1.08 | 1.11E-05 | 4530.OS02T0236000-01 4530.OS02T0600400-01 4530.OS03T0776000-01 4530.OS04T0390000-02 4530.OS06T0548000-01 4530.OS08T0536000-01 4530.OS09T0465600-01 4530.OS12T0616900-01 | OsJ_06017 OsJ_07404 OsJ_12779 OS04T0390000-02 OS06T0548000-01 OsJ_28086 OsJ_29674 OS12T0616900-01 |
| map03008 | Ribosome biogenesis in eukaryotes | 5 | 81 | 1.36 | 6.25E-05 | 4530.OS03T0300300-01 4530.OS04T0592700-01 4530.OS05T0176000-01 4530.OS07T0603200-01 4530.OS08T0158900-01 | OsJ_10517 OsJ_15986 OsJ_17309 OS07T0603200-01 OsJ_26117 |
| map00010 | Glycolysis / Gluconeogenesis | 5 | 127 | 1.17 | 0.00038 | 4530.OS03T0776000-01 4530.OS08T0536000-01 4530.OS09T0440300-01 4530.OS09T0465600-01 4530.OS12T0616900-01 | OsJ_12779 OsJ_28086 OS09T0440300-01 OsJ_29674 OS12T0616900-01 |
| map04712 | Circadian rhythm - plant | 3 | 27 | 1.62 | 0.00074 | 4530.OS01T0182600-03 4530.OS02T0625000-01 4530.OS02T0771100-01 | GI OsCRY2 PPS |
| map04120 | Ubiquitin mediated proteolysis | 4 | 108 | 1.14 | 0.0021 | 4530.OS02T0771100-01 4530.OS05T0557600-01 4530.OS07T0659800-01 4530.OS10T0438100-00 | PPS OsJ_19507 OS07T0659800-01 OsJ_31649 |
| map00330 | Arginine and proline metabolism | 3 | 48 | 1.37 | 0.0026 | 4530.OS02T0236000-01 4530.OS06T0548000-01 4530.OS09T0440300-01 | OsJ_06017 OS06T0548000-01 OS09T0440300-01 |
| map00020 | Citrate cycle (TCA cycle) | 3 | 53 | 1.32 | 0.003 | 4530.OS04T0390000-02 4530.OS08T0536000-01 4530.OS12T0616900-01 | OS04T0390000-02 OsJ_28086 OS12T0616900-01 |
| map00030 | Pentose phosphate pathway | 3 | 56 | 1.3 | 0.0031 | 4530.OS02T0600400-01 4530.OS03T0776000-01 4530.OS09T0465600-01 | OsJ_07404 OsJ_12779 OsJ_29674 |
| map00970 | Aminoacyl-tRNA biosynthesis | 3 | 56 | 1.3 | 0.0031 | 4530.OS01T0687500-01 4530.OS09T0556500-01 4530.OS10T0400100-01 | OsJ_03057 OsJ_30298 OsJ_31437 |
| map01110 | Biosynthesis of secondary metabolites | 9 | 943 | 0.55 | 0.0049 | 4530.OS02T0236000-01 4530.OS02T0600400-01 4530.OS03T0776000-01 4530.OS04T0390000-02 4530.OS06T0548000-01 4530.OS08T0536000-01 4530.OS09T0440300-01 4530.OS09T0465600-01 4530.OS12T0616900-01 | OsJ_06017 OsJ_07404 OsJ_12779 OS04T0390000-02 OS06T0548000-01 OsJ_28086 OS09T0440300-01 OsJ_29674 OS12T0616900-01 |
| map00620 | Pyruvate metabolism | 3 | 80 | 1.14 | 0.0062 | 4530.OS08T0536000-01 4530.OS09T0440300-01 4530.OS12T0616900-01 | OsJ_28086 OS09T0440300-01 OS12T0616900-01 |
| map00950 | Isoquinoline alkaloid biosynthesis | 2 | 20 | 1.57 | 0.0062 | 4530.OS02T0236000-01 4530.OS06T0548000-01 | OsJ_06017 OS06T0548000-01 |
| map00960 | Tropane piperidine and pyridine alkaloid biosynthesis | 2 | 19 | 1.59 | 0.0062 | 4530.OS02T0236000-01 4530.OS06T0548000-01 | OsJ_06017 OS06T0548000-01 |
| map00220 | Arginine biosynthesis | 2 | 31 | 1.38 | 0.0121 | 4530.OS02T0236000-01 4530.OS06T0548000-01 | OsJ_06017 OS06T0548000-01 |
| map00310 | Lysine degradation | 2 | 33 | 1.35 | 0.0128 | 4530.OS04T0390000-02 4530.OS09T0440300-01 | OS04T0390000-02 OS09T0440300-01 |
| map00350 | Tyrosine metabolism | 2 | 38 | 1.29 | 0.0156 | 4530.OS02T0236000-01 4530.OS06T0548000-01 | OsJ_06017 OS06T0548000-01 |
| map00380 | Tryptophan metabolism | 2 | 38 | 1.29 | 0.0156 | 4530.OS04T0390000-02 4530.OS09T0440300-01 | OS04T0390000-02 OS09T0440300-01 |
| map00360 | Phenylalanine metabolism | 2 | 45 | 1.22 | 0.0191 | 4530.OS02T0236000-01 4530.OS06T0548000-01 | OsJ_06017 OS06T0548000-01 |
| map00400 | Phenylalanine tyrosine and tryptophan biosynthesis | 2 | 46 | 1.21 | 0.0191 | 4530.OS02T0236000-01 4530.OS06T0548000-01 | OsJ_06017 OS06T0548000-01 |
| map00250 | Alanine aspartate and glutamate metabolism | 2 | 48 | 1.19 | 0.0195 | 4530.OS02T0236000-01 4530.OS06T0548000-01 | OsJ_06017 OS06T0548000-01 |
| map01210 | 2-Oxocarboxylic acid metabolism | 2 | 52 | 1.16 | 0.0216 | 4530.OS02T0236000-01 4530.OS06T0548000-01 | OsJ_06017 OS06T0548000-01 |
| map03040 | Spliceosome | 3 | 179 | 0.79 | 0.0291 | 4530.OS03T0339100-01 4530.OS06T0187900-02 4530.OS07T0508000-01 | OS03T0339100-01 RSZ21A OS07T0508000-01 |
| map01100 | Metabolic pathways | 10 | 1677 | 0.35 | 0.0322 | 4530.OS02T0236000-01 4530.OS02T0600400-01 4530.OS03T0776000-01 4530.OS04T0390000-02 4530.OS05T0573300-01 4530.OS06T0548000-01 4530.OS08T0536000-01 4530.OS09T0440300-01 4530.OS09T0465600-01 4530.OS12T0616900-01 | OsJ_06017 OsJ_07404 OsJ_12779 OS04T0390000-02 OsJ_19625 OS06T0548000-01 OsJ_28086 OS09T0440300-01 OsJ_29674 OS12T0616900-01 |
| map00710 | Carbon fixation in photosynthetic organisms | 2 | 73 | 1.01 | 0.0355 | 4530.OS02T0236000-01 4530.OS06T0548000-01 | OsJ_06017 OS06T0548000-01 |

Table S6. Information of the Molecular Function (Gene Ontology) targeted by *Botrytis cinerea.*

| #term ID | term description | observed gene count | background gene count | strength | false discovery rate | matching proteins in your network (IDs) | matching proteins in your network (labels) |
| --- | --- | --- | --- | --- | --- | --- | --- |
| GO:0005524 | ATP binding | 3 | 39 | 1.13 | 0.0074 | 4081.Solyc07g056140.2.1 4081.Solyc07g065840.2.1 4081.Solyc08g043170.2.1 | LOC543968 HSC80 PRO2 |
| GO:0032555 | purine ribonucleotide binding | 4 | 47 | 1.18 | 0.0074 | 4081.Solyc05g023800.2.1 4081.Solyc07g056140.2.1 4081.Solyc07g065840.2.1 4081.Solyc08g043170.2.1 | RAN1 LOC543968 HSC80 PRO2 |
| GO:0035639 | purine ribonucleoside triphosphate binding | 4 | 47 | 1.18 | 0.0074 | 4081.Solyc05g023800.2.1 4081.Solyc07g056140.2.1 4081.Solyc07g065840.2.1 4081.Solyc08g043170.2.1 | RAN1 LOC543968 HSC80 PRO2 |
| GO:0008144 | drug binding | 3 | 53 | 1 | 0.0112 | 4081.Solyc07g056140.2.1 4081.Solyc07g065840.2.1 4081.Solyc08g043170.2.1 | LOC543968 HSC80 PRO2 |
| GO:0016772 | transferase activity transferring phosphorus-containing groups | 2 | 17 | 1.32 | 0.0135 | 4081.Solyc07g056140.2.1 4081.Solyc08g043170.2.1 | LOC543968 PRO2 |

Table S7. Information of the KEGG_Pathway targeted by *Botrytis cinerea.*

| #term ID | term description | observed gene count | background gene count | strength | false discovery rate | matching proteins in your network (IDs) | matching proteins in your network (labels) |
| --- | --- | --- | --- | --- | --- | --- | --- |
| sly01100 | Metabolic pathways | 47 | 2050 | 0.61 | 1.60E-14 | 4081.Solyc01g008110.2.1 4081.Solyc01g008330.2.1 4081.Solyc01g080460.2.1 4081.Solyc01g081520.2.1 4081.Solyc01g099220.2.1 4081.Solyc01g106780.2.1 4081.Solyc01g109790.2.1 4081.Solyc02g094160.1.1 4081.Solyc03g065340.2.1 4081.Solyc03g071590.2.1 4081.Solyc03g098600.2.1 4081.Solyc03g111850.2.1 4081.Solyc03g122310.2.1 4081.Solyc04g007550.2.1 4081.Solyc04g009030.2.1 4081.Solyc04g011350.2.1 4081.Solyc04g076880.2.1 4081.Solyc04g077440.2.1 4081.Solyc04g081400.2.1 4081.Solyc04g082390.2.1 4081.Solyc05g008460.2.1 4081.Solyc05g009530.2.1 4081.Solyc06g019170.2.1 4081.Solyc06g048730.2.1 4081.Solyc06g051930.2.1 4081.Solyc06g071700.1.1 4081.Solyc07g052350.2.1 4081.Solyc07g055060.2.1 4081.Solyc07g055840.2.1 4081.Solyc07g056140.2.1 4081.Solyc07g062650.2.1 4081.Solyc07g063140.1.1 4081.Solyc07g064280.2.1 4081.Solyc07g066610.2.1 4081.Solyc08g043170.2.1 4081.Solyc09g075450.2.1 4081.Solyc09g090700.1.1 4081.Solyc09g098590.2.1 4081.Solyc11g007020.1.1 4081.Solyc11g011330.1.1 4081.Solyc11g030600.2.1 4081.Solyc11g072520.1.1 4081.Solyc12g005080.1.1 4081.Solyc12g005630.1.1 4081.Solyc12g014250.1.1 4081.Solyc12g019180.1.1 4081.Solyc12g096730.1.1 | CYP51 101267921 101267346 101263844 Solyc01g099220.2.1 101259587 AGP-S1 101268630 101252851 chMDH 101259924 101250390 Solyc03g122310.2.1 101249170 101266713 101264112 LOC543812 Solyc04g077440.2.1 HXK4 101257856 101261919 101257857 101244293 101250511 101257775 101253199 101254008 101248407 101258079 LOC543968 mMDH GATC 101254921 101253805 PRO2 101265278 SSADH SUS4 MDC 101259903 MET Solyc11g072520.1.1 Solyc12g005080.1.1 101243864 ppc1 101260021 TPG6 |
| sly01200 | Carbon metabolism | 18 | 266 | 1.08 | 1.87E-12 | 4081.Solyc01g008330.2.1 4081.Solyc01g080460.2.1 4081.Solyc01g106780.2.1 4081.Solyc03g071590.2.1 4081.Solyc04g009030.2.1 4081.Solyc04g011350.2.1 4081.Solyc04g076880.2.1 4081.Solyc04g081400.2.1 4081.Solyc05g009530.2.1 4081.Solyc06g051930.2.1 4081.Solyc07g052350.2.1 4081.Solyc07g055060.2.1 4081.Solyc07g055840.2.1 4081.Solyc07g062650.2.1 4081.Solyc07g066610.2.1 4081.Solyc09g075450.2.1 4081.Solyc12g005080.1.1 4081.Solyc12g014250.1.1 | 101267921 101267346 101259587 chMDH 101266713 101264112 LOC543812 HXK4 101257857 101257775 101254008 101248407 101258079 mMDH 101253805 101265278 Solyc12g005080.1.1 ppc1 |
| sly00620 | Pyruvate metabolism | 12 | 91 | 1.37 | 1.67E-11 | 4081.Solyc01g008330.2.1 4081.Solyc01g080460.2.1 4081.Solyc01g106780.2.1 4081.Solyc03g071590.2.1 4081.Solyc03g122310.2.1 4081.Solyc04g076880.2.1 4081.Solyc05g009530.2.1 4081.Solyc06g051930.2.1 4081.Solyc07g055060.2.1 4081.Solyc07g062650.2.1 4081.Solyc09g075450.2.1 4081.Solyc12g014250.1.1 | 101267921 101267346 101259587 chMDH Solyc03g122310.2.1 LOC543812 101257857 101257775 101248407 mMDH 101265278 ppc1 |
| sly01110 | Biosynthesis of secondary metabolites | 28 | 1192 | 0.62 | 5.66E-09 | 4081.Solyc01g008110.2.1 4081.Solyc01g008330.2.1 4081.Solyc01g106780.2.1 4081.Solyc01g109790.2.1 4081.Solyc03g065340.2.1 4081.Solyc03g111850.2.1 4081.Solyc03g122310.2.1 4081.Solyc04g011350.2.1 4081.Solyc04g076880.2.1 4081.Solyc04g077440.2.1 4081.Solyc04g081400.2.1 4081.Solyc05g009530.2.1 4081.Solyc06g019170.2.1 4081.Solyc06g048730.2.1 4081.Solyc06g051930.2.1 4081.Solyc06g071700.1.1 4081.Solyc07g052350.2.1 4081.Solyc07g055840.2.1 4081.Solyc07g056140.2.1 4081.Solyc07g062650.2.1 4081.Solyc07g064280.2.1 4081.Solyc07g066610.2.1 4081.Solyc08g043170.2.1 4081.Solyc09g075450.2.1 4081.Solyc11g007020.1.1 4081.Solyc11g011330.1.1 4081.Solyc11g072520.1.1 4081.Solyc12g005080.1.1 | CYP51 101267921 101259587 AGP-S1 101252851 101250390 Solyc03g122310.2.1 101264112 LOC543812 Solyc04g077440.2.1 HXK4 101257857 101244293 101250511 101257775 101253199 101254008 101258079 LOC543968 mMDH 101254921 101253805 PRO2 101265278 MDC 101259903 Solyc11g072520.1.1 Solyc12g005080.1.1 |
| sly00020 | Citrate cycle (TCA cycle) | 8 | 54 | 1.42 | 3.19E-08 | 4081.Solyc04g011350.2.1 4081.Solyc04g076880.2.1 4081.Solyc05g009530.2.1 4081.Solyc07g052350.2.1 4081.Solyc07g055840.2.1 4081.Solyc07g062650.2.1 4081.Solyc09g075450.2.1 4081.Solyc12g005080.1.1 | 101264112 LOC543812 101257857 101254008 101258079 mMDH 101265278 Solyc12g005080.1.1 |
| sly00710 | Carbon fixation in photosynthetic organisms | 8 | 76 | 1.27 | 3.08E-07 | 4081.Solyc01g080460.2.1 4081.Solyc03g071590.2.1 4081.Solyc04g009030.2.1 4081.Solyc04g076880.2.1 4081.Solyc07g055060.2.1 4081.Solyc07g062650.2.1 4081.Solyc07g066610.2.1 4081.Solyc12g014250.1.1 | 101267346 chMDH 101266713 LOC543812 101248407 mMDH 101253805 ppc1 |
| sly03008 | Ribosome biogenesis in eukaryotes | 7 | 75 | 1.22 | 4.26E-06 | 4081.Solyc01g109990.2.1 4081.Solyc03g046350.2.1 4081.Solyc03g116380.2.1 4081.Solyc04g051670.2.1 4081.Solyc04g064570.2.1 4081.Solyc04g072260.1.1 4081.Solyc05g023800.2.1 | 101263645 101259128 101255780 101258943 101258649 MDN1 RAN1 |
| sly01230 | Biosynthesis of amino acids | 10 | 232 | 0.88 | 1.11E-05 | 4081.Solyc01g106780.2.1 4081.Solyc03g111850.2.1 4081.Solyc06g019170.2.1 4081.Solyc06g051930.2.1 4081.Solyc07g052350.2.1 4081.Solyc07g055840.2.1 4081.Solyc07g064280.2.1 4081.Solyc07g066610.2.1 4081.Solyc08g043170.2.1 4081.Solyc11g072520.1.1 | 101259587 101250390 101244293 101257775 101254008 101258079 101254921 101253805 PRO2 Solyc11g072520.1.1 |
| sly03010 | Ribosome | 11 | 290 | 0.82 | 1.11E-05 | 4081.Solyc01g009520.2.1 4081.Solyc03g019780.2.1 4081.Solyc04g008810.2.1 4081.Solyc05g054070.2.1 4081.Solyc05g054990.2.1 4081.Solyc06g007470.2.1 4081.Solyc06g051200.2.1 4081.Solyc06g064460.2.1 4081.Solyc06g064470.2.1 4081.Solyc07g041310.2.1 4081.Solyc08g067950.2.1 | 101245686 101250574 101247310 101250600 101251184 101248302 101247532 101245956 101246256 101257282 101252799 |
| sly00010 | Glycolysis / Gluconeogenesis | 7 | 132 | 0.97 | 0.0001 | 4081.Solyc01g106780.2.1 4081.Solyc03g122310.2.1 4081.Solyc04g076880.2.1 4081.Solyc04g081400.2.1 4081.Solyc05g009530.2.1 4081.Solyc06g051930.2.1 4081.Solyc07g066610.2.1 | 101259587 Solyc03g122310.2.1 LOC543812 HXK4 101257857 101257775 101253805 |
| sly03013 | RNA transport | 7 | 159 | 0.89 | 0.00029 | 4081.Solyc01g095410.2.1 4081.Solyc01g098170.2.1 4081.Solyc05g023800.2.1 4081.Solyc08g007110.2.1 4081.Solyc08g062800.2.1 4081.Solyc09g010460.2.1 4081.Solyc10g047000.1.1 | 101248917 101250741 RAN1 101258879 101252510 Solyc09g010460.2.1 101247869 |
| sly03015 | mRNA surveillance pathway | 6 | 124 | 0.93 | 0.00057 | 4081.Solyc01g099860.2.1 4081.Solyc02g021760.2.1 4081.Solyc08g007110.2.1 4081.Solyc08g067040.2.1 4081.Solyc12g035360.1.1 4081.Solyc12g099280.1.1 | 101258308 101263950 101258879 Solyc08g067040.2.1 Solyc12g035360.1.1 101259137 |
| sly03040 | Spliceosome | 7 | 182 | 0.83 | 0.00057 | 4081.Solyc01g068480.2.1 4081.Solyc01g103450.2.1 4081.Solyc01g110990.2.1 4081.Solyc04g008890.2.1 4081.Solyc04g014210.2.1 4081.Solyc07g008880.2.1 4081.Solyc08g069080.2.1 | 101244224 101265681 Solyc01g110990.2.1 101244858 101247309 101261833 101247946 |
| sly00500 | Starch and sucrose metabolism | 6 | 132 | 0.9 | 0.00068 | 4081.Solyc01g081520.2.1 4081.Solyc01g109790.2.1 4081.Solyc03g065340.2.1 4081.Solyc04g081400.2.1 4081.Solyc07g056140.2.1 4081.Solyc09g098590.2.1 | 101263844 AGP-S1 101252851 HXK4 LOC543968 SUS4 |
| sly00970 | Aminoacyl-tRNA biosynthesis | 4 | 51 | 1.14 | 0.0011 | 4081.Solyc01g080810.2.1 4081.Solyc01g109360.2.1 4081.Solyc01g111990.2.1 4081.Solyc07g063140.1.1 | 101249760 Solyc01g109360.2.1 101251229 GATC |
| sly03050 | Proteasome | 4 | 49 | 1.16 | 0.0011 | 4081.Solyc05g018570.2.1 4081.Solyc05g018590.2.1 4081.Solyc07g066030.2.1 4081.Solyc10g008010.2.1 | 101245359 101245649 101266536 101245889 |
| sly00330 | Arginine and proline metabolism | 4 | 65 | 1.03 | 0.0026 | 4081.Solyc03g118690.2.1 4081.Solyc03g122310.2.1 4081.Solyc06g019170.2.1 4081.Solyc08g043170.2.1 | 101265811 Solyc03g122310.2.1 101244293 PRO2 |
| sly00630 | Glyoxylate and dicarboxylate metabolism | 4 | 74 | 0.98 | 0.0038 | 4081.Solyc06g060770.2.1 4081.Solyc07g052350.2.1 4081.Solyc07g055840.2.1 4081.Solyc07g062650.2.1 | 101251299 101254008 101258079 mMDH |
| sly00310 | Lysine degradation | 3 | 39 | 1.13 | 0.0062 | 4081.Solyc03g122310.2.1 4081.Solyc04g011350.2.1 4081.Solyc12g005080.1.1 | Solyc03g122310.2.1 101264112 Solyc12g005080.1.1 |
| sly03030 | DNA replication | 3 | 46 | 1.06 | 0.0092 | 4081.Solyc01g079500.2.1 4081.Solyc01g110130.2.1 4081.Solyc06g060150.2.1 | 101243636 101264867 101264020 |
| sly00380 | Tryptophan metabolism | 3 | 48 | 1.04 | 0.0098 | 4081.Solyc03g118690.2.1 4081.Solyc03g122310.2.1 4081.Solyc04g011350.2.1 | 101265811 Solyc03g122310.2.1 101264112 |
| sly00400 | Phenylalanine tyrosine and tryptophan biosynthesis | 3 | 48 | 1.04 | 0.0098 | 4081.Solyc03g111850.2.1 4081.Solyc07g064280.2.1 4081.Solyc11g072520.1.1 | 101250390 101254921 Solyc11g072520.1.1 |
| sly00520 | Amino sugar and nucleotide sugar metabolism | 4 | 132 | 0.73 | 0.0223 | 4081.Solyc01g081520.2.1 4081.Solyc01g109790.2.1 4081.Solyc04g081400.2.1 4081.Solyc07g056140.2.1 | 101263844 AGP-S1 HXK4 LOC543968 |
| sly00260 | Glycine serine and threonine metabolism | 3 | 69 | 0.88 | 0.0225 | 4081.Solyc03g122310.2.1 4081.Solyc06g071700.1.1 4081.Solyc07g064280.2.1 | Solyc03g122310.2.1 101253199 101254921 |
| sly04141 | Protein processing in endoplasmic reticulum | 5 | 213 | 0.62 | 0.0225 | 4081.Solyc01g088020.2.1 4081.Solyc01g103450.2.1 4081.Solyc03g082920.2.1 4081.Solyc03g098600.2.1 4081.Solyc07g065840.2.1 | 101261454 101265681 Solyc03g082920.2.1 101259924 HSC80 |
| sly04120 | Ubiquitin mediated proteolysis | 4 | 142 | 0.7 | 0.0252 | 4081.Solyc03g095210.1.1 4081.Solyc04g076620.2.1 4081.Solyc07g065630.2.1 4081.Solyc10g055450.1.1 | 101251072 Solyc04g076620.2.1 Solyc07g065630.2.1 101247472 |
| sly00230 | Purine metabolism | 4 | 159 | 0.65 | 0.0352 | 4081.Solyc01g106780.2.1 4081.Solyc02g032550.1.1 4081.Solyc02g094160.1.1 4081.Solyc06g051930.2.1 | 101259587 Solyc02g032550.1.1 101268630 101257775 |
| sly04136 | Autophagy - other | 2 | 35 | 1 | 0.0451 | 4081.Solyc01g106770.2.1 4081.Solyc04g012170.2.1 | Solyc01g106770.2.1 Solyc04g012170.2.1 |
| sly00100 | Steroid biosynthesis | 2 | 36 | 0.99 | 0.0458 | 4081.Solyc01g008110.2.1 4081.Solyc04g077440.2.1 | CYP51 Solyc04g077440.2.1 |

Table S8. Information of the KEGG_Pathway targeted by *Phytophthora infestans*.

| #term ID | term description | observed gene count | background gene count | strength | false discovery rate | matching proteins in your network (IDs) | matching proteins in your network (labels) |
| --- | --- | --- | --- | --- | --- | --- | --- |
| sot01100 | Metabolic pathways | 23 | 1851 | 0.64 | 1.46E-07 | 4113.PGSC0003DMT400002595 4113.PGSC0003DMT400004095 4113.PGSC0003DMT400006578 4113.PGSC0003DMT400011868 4113.PGSC0003DMT400012593 4113.PGSC0003DMT400023090 4113.PGSC0003DMT400028915 4113.PGSC0003DMT400035467 4113.PGSC0003DMT400035987 4113.PGSC0003DMT400036942 4113.PGSC0003DMT400042035 4113.PGSC0003DMT400063336 4113.PGSC0003DMT400065000 4113.PGSC0003DMT400066112 4113.PGSC0003DMT400069311 4113.PGSC0003DMT400069799 4113.PGSC0003DMT400070265 4113.PGSC0003DMT400072632 4113.PGSC0003DMT400072658 4113.PGSC0003DMT400076178 4113.PGSC0003DMT400076179 4113.PGSC0003DMT400077231 4113.PGSC0003DMT400079915 | PGSC0003DMT400002595 102598888 102598451 102582524 102579136 InvGF PGSC0003DMT400028915 102597065 Pain-1 102580238 102603859 102577433 107058768 102604947 102594988 102599296 102582462 PGSC0003DMT400072632 102592163 CHS1B 102580017 102582317 PGSC0003DMT400079915 |
| sot03010 | Ribosome | 10 | 269 | 1.11 | 2.16E-07 | 4113.PGSC0003DMT400010548 4113.PGSC0003DMT400017986 4113.PGSC0003DMT400030564 4113.PGSC0003DMT400036835 4113.PGSC0003DMT400048186 4113.PGSC0003DMT400053000 4113.PGSC0003DMT400059174 4113.PGSC0003DMT400060177 4113.PGSC0003DMT400069575 4113.PGSC0003DMT400079312 | 102582667 102587574 102588127 102579491 102596272 102580154 PGSC0003DMT400059174 102590182 102590512 102605043 |
| sot01200 | Carbon metabolism | 9 | 255 | 1.09 | 1.26E-06 | 4113.PGSC0003DMT400006578 4113.PGSC0003DMT400011868 4113.PGSC0003DMT400028915 4113.PGSC0003DMT400065000 4113.PGSC0003DMT400066112 4113.PGSC0003DMT400069799 4113.PGSC0003DMT400070265 4113.PGSC0003DMT400072632 4113.PGSC0003DMT400072658 | 102598451 102582524 PGSC0003DMT400028915 107058768 102604947 102599296 102582462 PGSC0003DMT400072632 102592163 |
| sot03008 | Ribosome biogenesis in eukaryotes | 6 | 73 | 1.46 | 1.41E-06 | 4113.PGSC0003DMT400007765 4113.PGSC0003DMT400017459 4113.PGSC0003DMT400032418 4113.PGSC0003DMT400062992 4113.PGSC0003DMT400066026 4113.PGSC0003DMT400093989 | 102580337 102578407 102585048 102603701 102603891 102603390 |
| sot03013 | RNA transport | 7 | 140 | 1.24 | 2.38E-06 | 4113.PGSC0003DMT400004341 4113.PGSC0003DMT400029432 4113.PGSC0003DMT400060512 4113.PGSC0003DMT400068044 4113.PGSC0003DMT400078176 4113.PGSC0003DMT400078293 4113.PGSC0003DMT400079119 | 102589798 102585342 102588992 102594613 102595553 102605977 102604773 |
| sot00052 | Galactose metabolism | 5 | 50 | 1.54 | 4.54E-06 | 4113.PGSC0003DMT400023090 4113.PGSC0003DMT400035987 4113.PGSC0003DMT400039505 4113.PGSC0003DMT400065096 4113.PGSC0003DMT400079915 | InvGF Pain-1 102601840 102601663 PGSC0003DMT400079915 |
| sot00010 | Glycolysis / Gluconeogenesis | 6 | 113 | 1.27 | 9.15E-06 | 4113.PGSC0003DMT400028915 4113.PGSC0003DMT400066112 4113.PGSC0003DMT400069799 4113.PGSC0003DMT400070265 4113.PGSC0003DMT400072632 4113.PGSC0003DMT400077231 | PGSC0003DMT400028915 102604947 102599296 102582462 PGSC0003DMT400072632 102582317 |
| sot00260 | Glycine serine and threonine metabolism | 5 | 70 | 1.4 | 1.61E-05 | 4113.PGSC0003DMT400006578 4113.PGSC0003DMT400011868 4113.PGSC0003DMT400065000 4113.PGSC0003DMT400069799 4113.PGSC0003DMT400072658 | 102598451 102582524 107058768 102599296 102592163 |
| sot01110 | Biosynthesis of secondary metabolites | 14 | 1083 | 0.65 | 1.78E-05 | 4113.PGSC0003DMT400002595 4113.PGSC0003DMT400004095 4113.PGSC0003DMT400006578 4113.PGSC0003DMT400011868 4113.PGSC0003DMT400012593 4113.PGSC0003DMT400036942 4113.PGSC0003DMT400065000 4113.PGSC0003DMT400066112 4113.PGSC0003DMT400069799 4113.PGSC0003DMT400070265 4113.PGSC0003DMT400072632 4113.PGSC0003DMT400072658 4113.PGSC0003DMT400076178 4113.PGSC0003DMT400076179 | PGSC0003DMT400002595 102598888 102598451 102582524 102579136 102580238 107058768 102604947 102599296 102582462 PGSC0003DMT400072632 102592163 CHS1B 102580017 |
| sot01230 | Biosynthesis of amino acids | 6 | 210 | 1 | 0.00019 | 4113.PGSC0003DMT400011868 4113.PGSC0003DMT400065000 4113.PGSC0003DMT400066112 4113.PGSC0003DMT400070265 4113.PGSC0003DMT400072632 4113.PGSC0003DMT400072658 | 102582524 107058768 102604947 102582462 PGSC0003DMT400072632 102592163 |
| sot00230 | Purine metabolism | 5 | 140 | 1.09 | 0.00029 | 4113.PGSC0003DMT400002595 4113.PGSC0003DMT400004095 4113.PGSC0003DMT400042035 4113.PGSC0003DMT400066112 4113.PGSC0003DMT400070265 | PGSC0003DMT400002595 102598888 102603859 102604947 102582462 |
| sot00600 | Sphingolipid metabolism | 3 | 26 | 1.6 | 0.00033 | 4113.PGSC0003DMT400039505 4113.PGSC0003DMT400065096 4113.PGSC0003DMT400069311 | 102601840 102601663 102594988 |
| sot00630 | Glyoxylate and dicarboxylate metabolism | 4 | 77 | 1.26 | 0.00034 | 4113.PGSC0003DMT400006578 4113.PGSC0003DMT400011868 4113.PGSC0003DMT400065000 4113.PGSC0003DMT400069799 | 102598451 102582524 107058768 102599296 |
| sot00603 | Glycosphingolipid biosynthesis - globo and isoglobo series | 2 | 7 | 2 | 0.00099 | 4113.PGSC0003DMT400039505 4113.PGSC0003DMT400065096 | 102601840 102601663 |
| sot00970 | Aminoacyl-tRNA biosynthesis | 3 | 41 | 1.41 | 0.00099 | 4113.PGSC0003DMT400024220 4113.PGSC0003DMT400026761 4113.PGSC0003DMT400059195 | PGSC0003DMT400024220 102606382 102604688 |
| sot00561 | Glycerolipid metabolism | 3 | 74 | 1.15 | 0.0045 | 4113.PGSC0003DMT400006578 4113.PGSC0003DMT400039505 4113.PGSC0003DMT400065096 | 102598451 102601840 102601663 |
| sot00670 | One carbon pool by folate | 2 | 20 | 1.54 | 0.0053 | 4113.PGSC0003DMT400011868 4113.PGSC0003DMT400065000 | 102582524 107058768 |
| sot00620 | Pyruvate metabolism | 3 | 83 | 1.1 | 0.0055 | 4113.PGSC0003DMT400066112 4113.PGSC0003DMT400069799 4113.PGSC0003DMT400070265 | 102604947 102599296 102582462 |
| sot00500 | Starch and sucrose metabolism | 3 | 111 | 0.97 | 0.0115 | 4113.PGSC0003DMT400023090 4113.PGSC0003DMT400035987 4113.PGSC0003DMT400079915 | InvGF Pain-1 PGSC0003DMT400079915 |
| sot03430 | Mismatch repair | 2 | 34 | 1.31 | 0.012 | 4113.PGSC0003DMT400010422 4113.PGSC0003DMT400078207 | 102598532 102581875 |
| sot03060 | Protein export | 2 | 37 | 1.27 | 0.0134 | 4113.PGSC0003DMT400031635 4113.PGSC0003DMT400070672 | 102601905 102585784 |
| sot00460 | Cyanoamino acid metabolism | 2 | 40 | 1.24 | 0.0141 | 4113.PGSC0003DMT400011868 4113.PGSC0003DMT400065000 | 102582524 107058768 |
| sot04712 | Circadian rhythm - plant | 2 | 39 | 1.25 | 0.0141 | 4113.PGSC0003DMT400076178 4113.PGSC0003DMT400076179 | CHS1B 102580017 |
| sot00941 | Flavonoid biosynthesis | 2 | 49 | 1.15 | 0.0197 | 4113.PGSC0003DMT400076178 4113.PGSC0003DMT400076179 | CHS1B 102580017 |
| sot00030 | Pentose phosphate pathway | 2 | 52 | 1.13 | 0.0211 | 4113.PGSC0003DMT400028915 4113.PGSC0003DMT400072632 | PGSC0003DMT400028915 PGSC0003DMT400072632 |
| sot03040 | Spliceosome | 3 | 175 | 0.78 | 0.0282 | 4113.PGSC0003DMT400056692 4113.PGSC0003DMT400069042 4113.PGSC0003DMT400090250 | 102590563 102604583 102590431 |

# Supplementary Figures

Figure S1. *Magnaporthe oryzae* targeting *Oryza sativa* KEGG_Pathway bubble diagram.


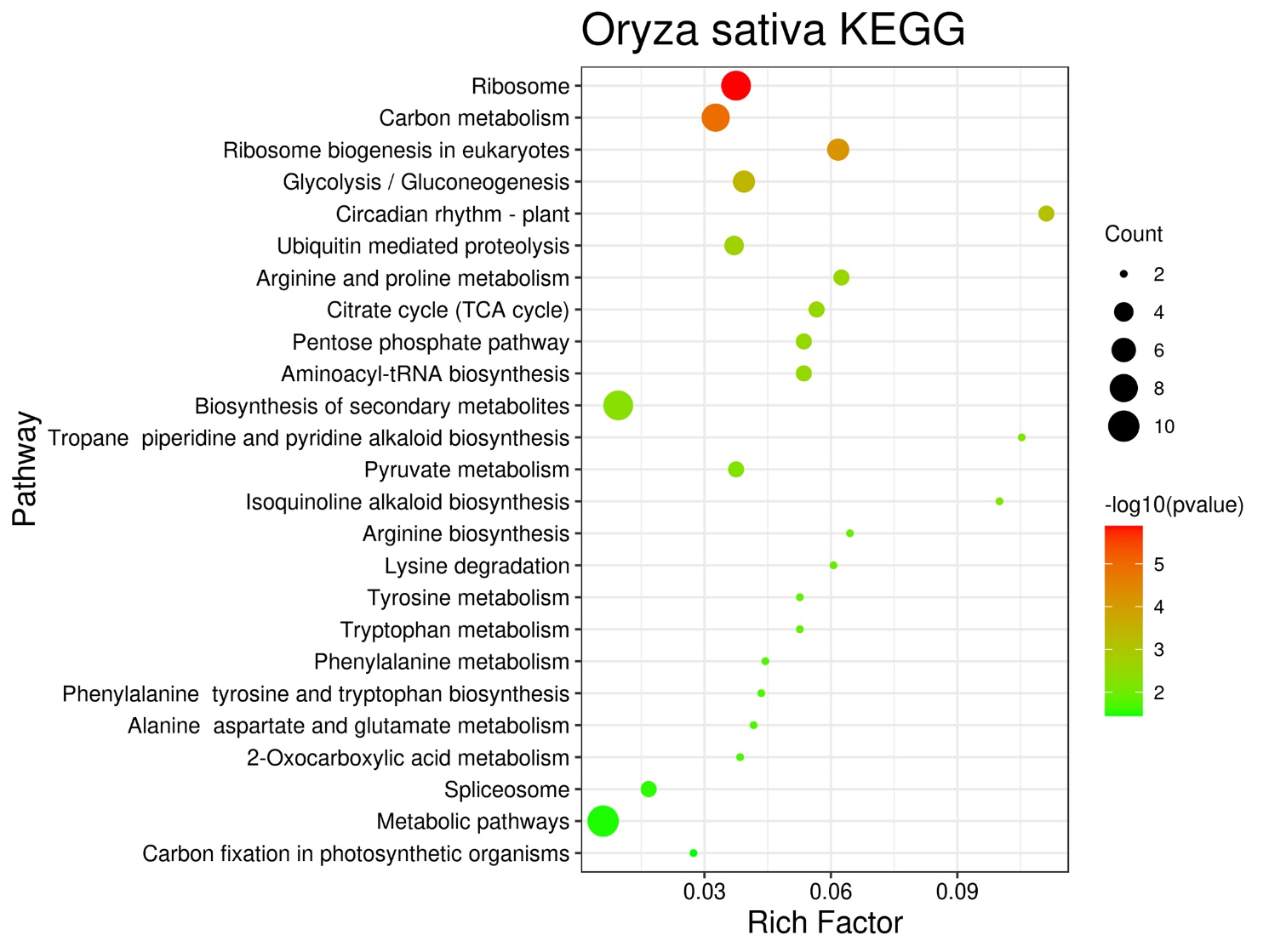


Figure S2. Bubble diagram of the KEGG_pathway of *Phytophthora infestans* targeting *Solanum tuberosum*.


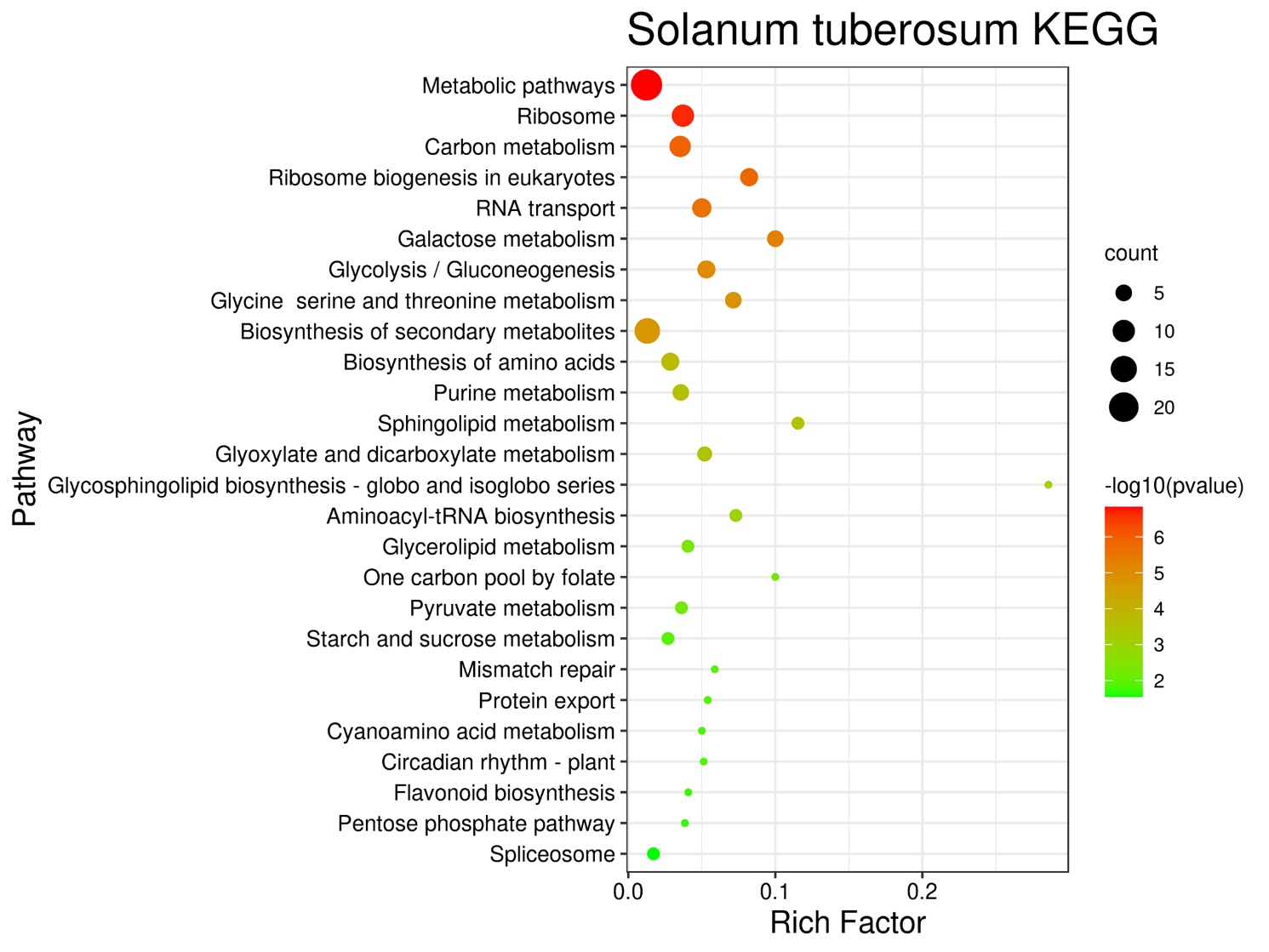


Figure S3. Evaluation of five models of *Magnaporthe oryzae*.


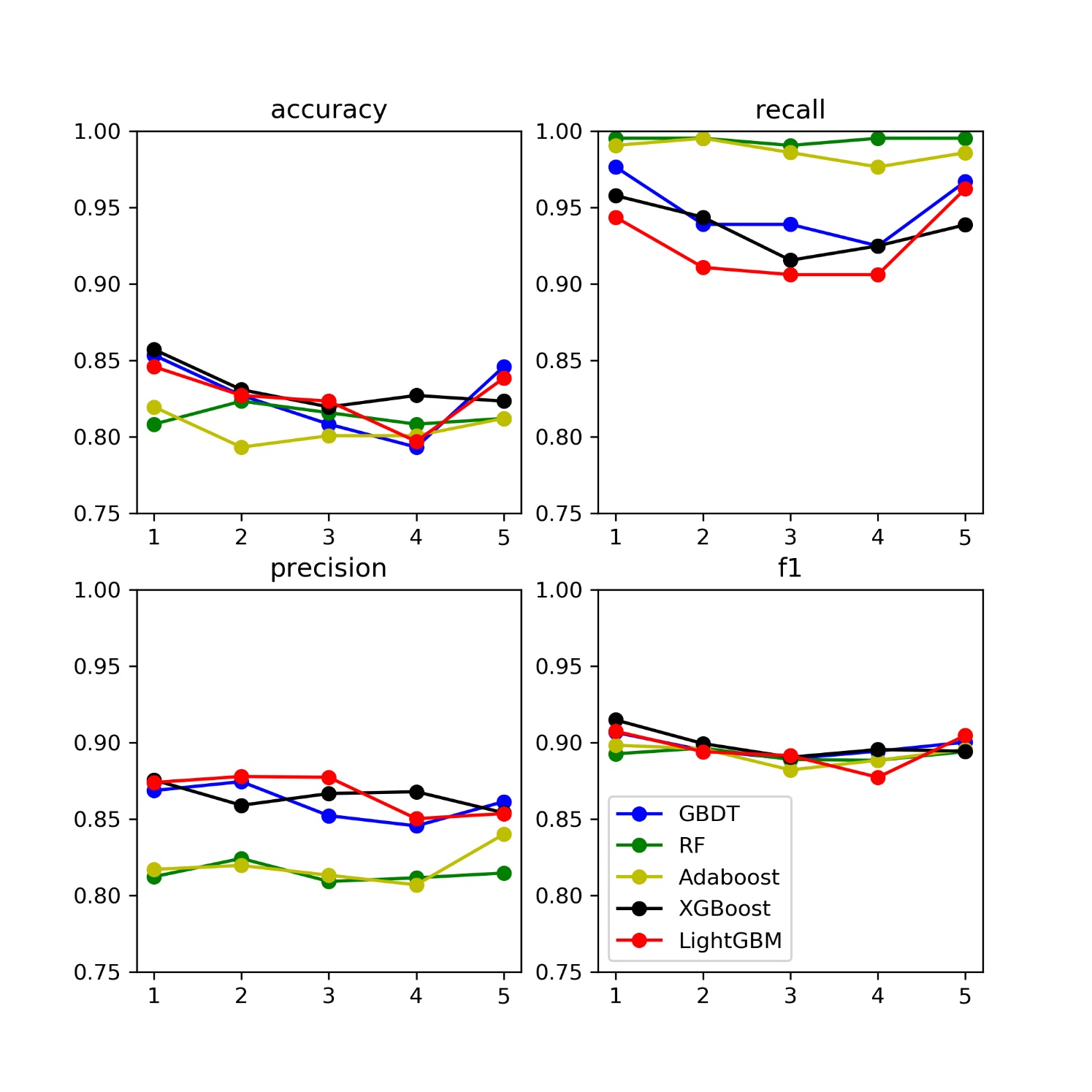


Figure S4. Evaluation of five models of *Phytophthora infestans*.


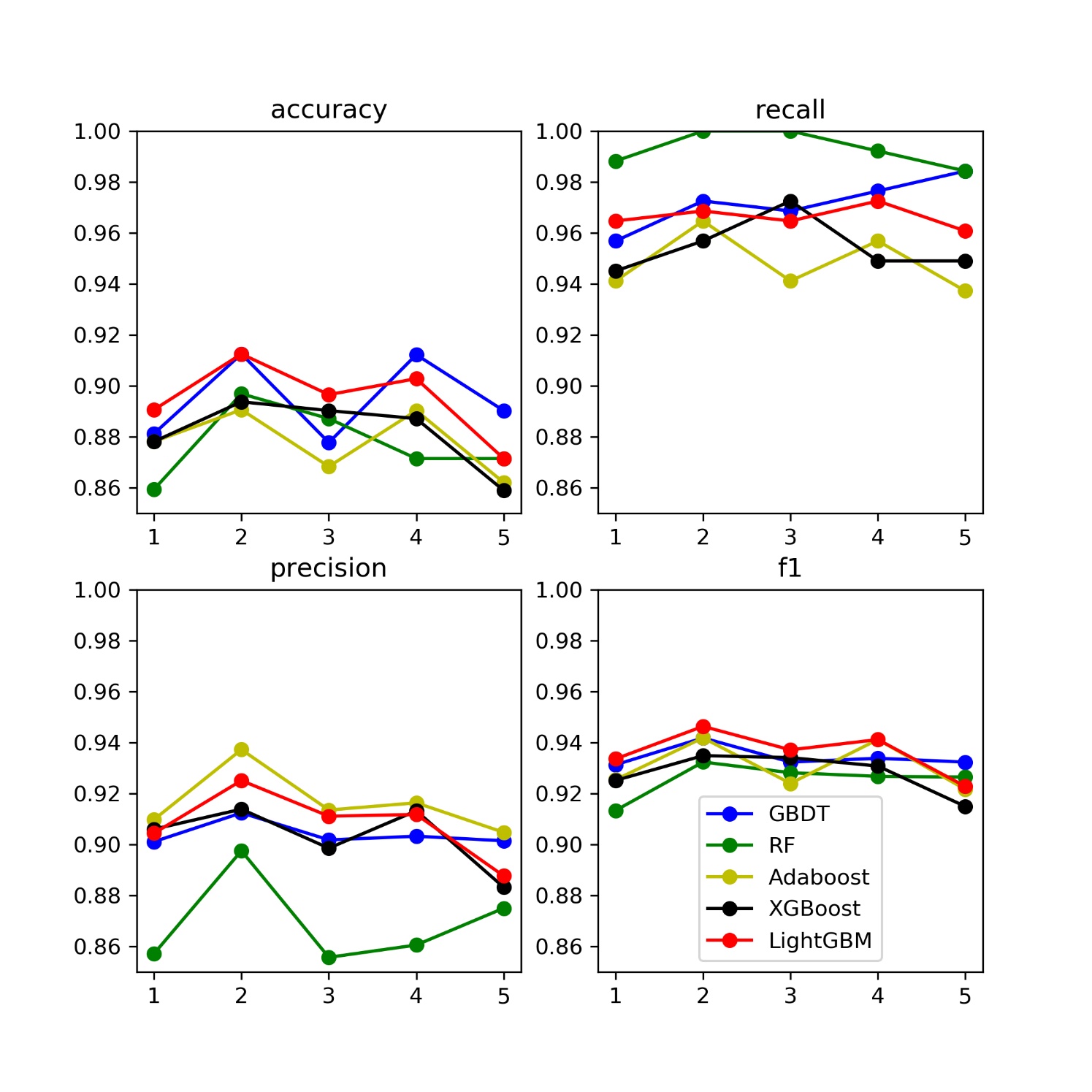


Figure S5. ROC curves of five models of *Magnaporthe oryzae*.


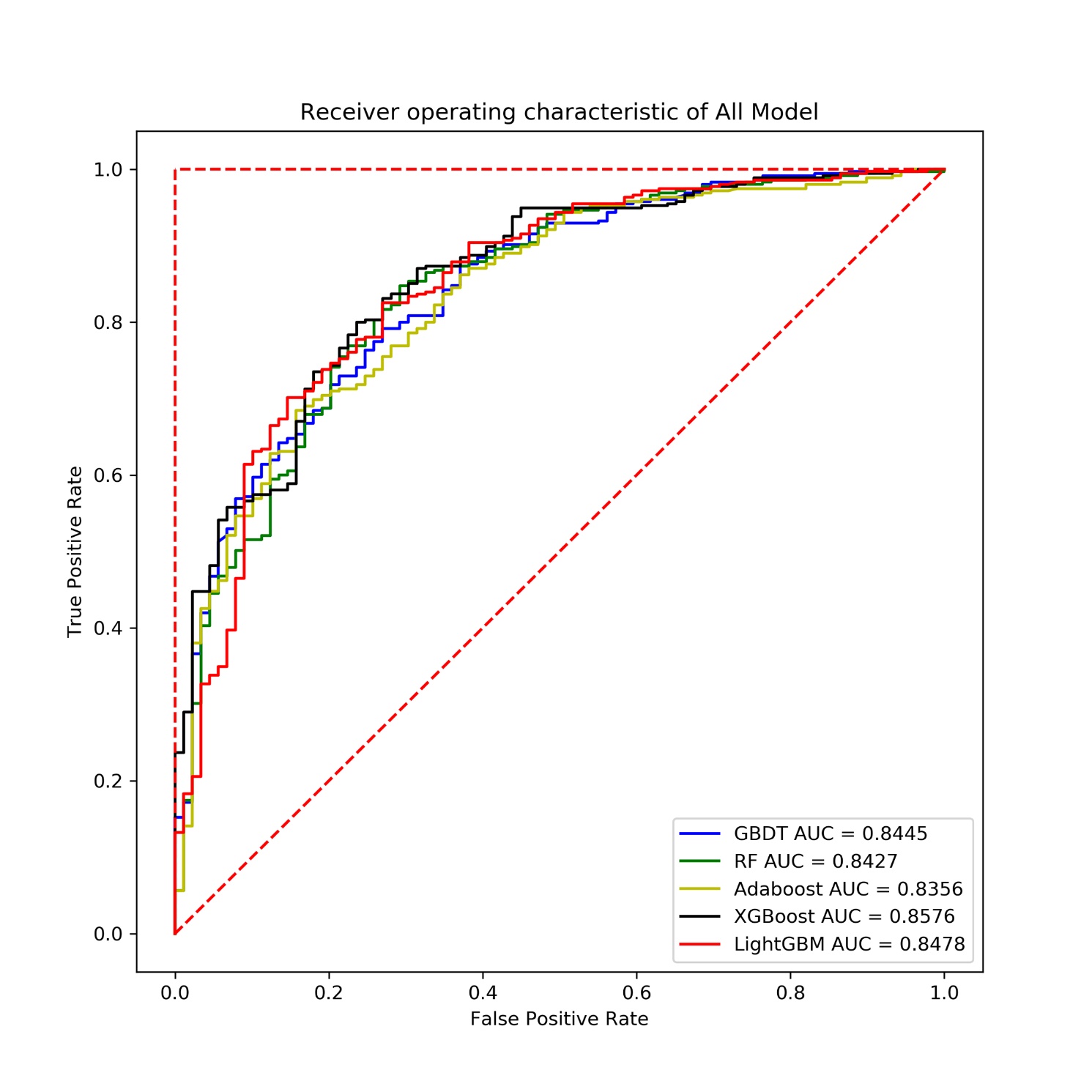


Figure S6. ROC curves of five models of *Phytophthora infestans*.


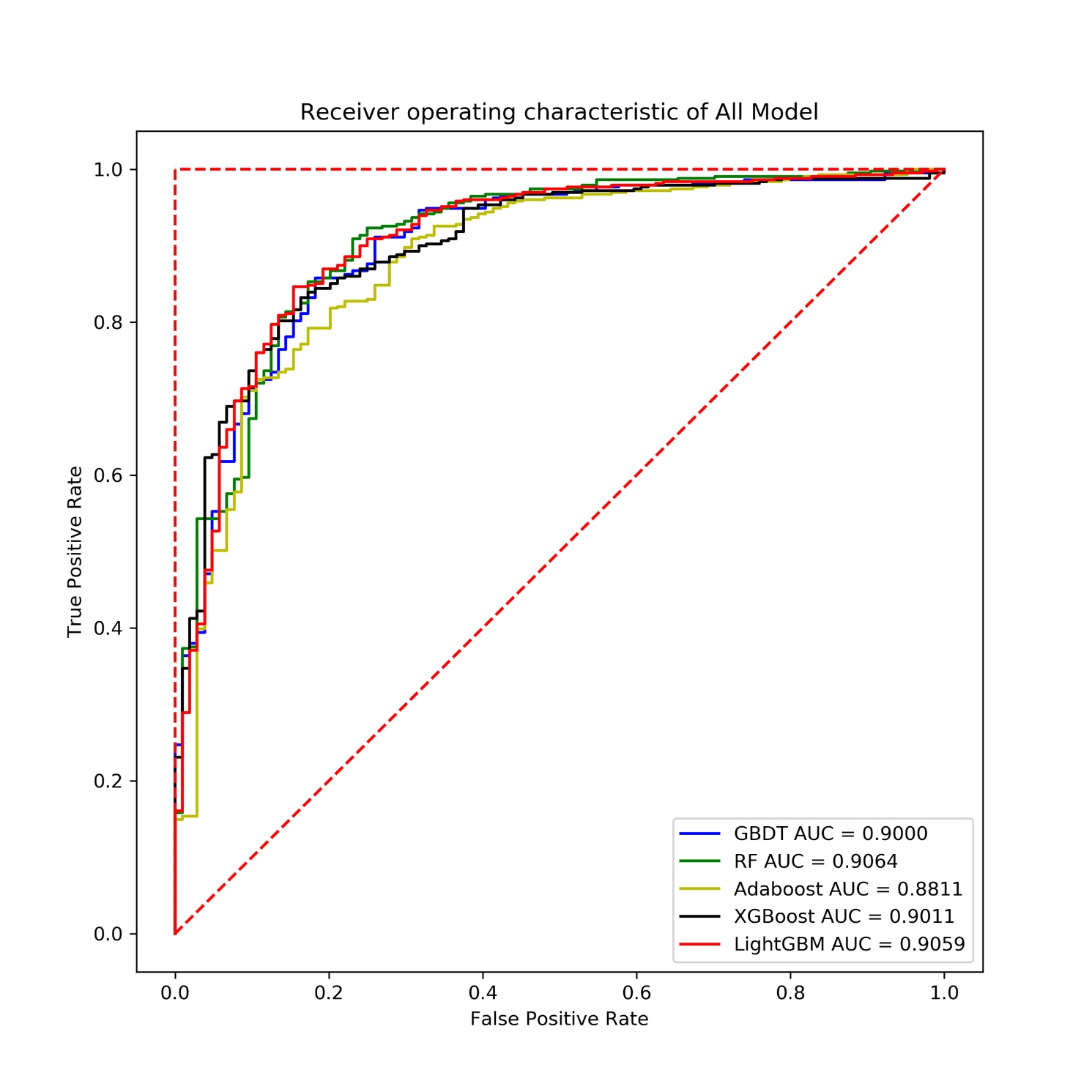

Supplement: Supplementary file 1 [file DataSheet1.docx]
